# Impaired pre-synaptic plasticity and visual responses in auxilin-knockout mice

**DOI:** 10.1016/j.isci.2023.107842

**Published:** 2023-09-06

**Authors:** Xi Cheng, Yu Tang, D.J. Vidyadhara, Ben-Zheng Li, Michael Zimmerman, Alexandr Pak, Sanghamitra Nareddula, Paige Alyssa Edens, Sreeganga S. Chandra, Alexander A. Chubykin

**Affiliations:** 1 Department of Biological Sciences, Purdue Institute for Integrative Neuroscience, Purdue Autism Research Center, Purdue University, West Lafayette, IN 47907, USA; 2 Department of Neurology, Yale University, CT, USA; 3 Department of Neuroscience, Yale University, CT, USA; 4 Department of Physiology and Biophysics, University of Colorado Anschutz Medical Campus, Aurora, CO, USA; 5 Department of Electrical Engineering, University of Colorado, Denver, Denver, CO, USA; 6 Department of Biomedical Engineering, Purdue University, West Lafayette, IN 47907, USA; 7 Program in Cellular Neuroscience, Neurodegeneration and Repair, Yale University, CT, USA

**Keywords:** Natural sciences, Biological sciences, Physiology, Neuroscience

## Abstract

Auxilin (DNAJC6/PARK19), an endocytic co-chaperone, is essential for maintaining homeostasis in the readily releasable pool (RRP) by aiding clathrin-mediated uncoating of synaptic vesicles. Its loss-of-function mutations, observed in familial Parkinson’s disease (PD), lead to basal ganglia motor deficits and cortical dysfunction. We discovered that auxilin-knockout (Aux-KO) mice exhibited impaired pre-synaptic plasticity in layer 4 to layer 2/3 pyramidal cell synapses in the primary visual cortex (V1), including reduced short-term facilitation and depression. Computational modeling revealed increased RRP refilling during short repetitive stimulation, which diminished during prolonged stimulation. Silicon probe recordings in V1 of Aux-KO mice demonstrated disrupted visual cortical circuit responses, including reduced orientation selectivity, compromised visual mismatch negativity, and shorter visual familiarity-evoked theta oscillations. Pupillometry analysis revealed an impaired optokinetic response. Auxilin-dependent pre-synaptic endocytosis dysfunction was associated with deficits in pre-synaptic plasticity, visual cortical functions, and eye movement prodromally or at the early stage of motor symptoms.

## Highlights

• Pre-synaptic short-term plasticity is impaired in Aux-KO mice
• Orientation selectivity and visual mismatch responses are reduced in Aux-KO mice
• Visual familiarity-induced oscillations are reduced in Aux-KO mice
• Optokinetic responses are impaired in Aux-KO mice

## Graphical abstract

**Figure:**
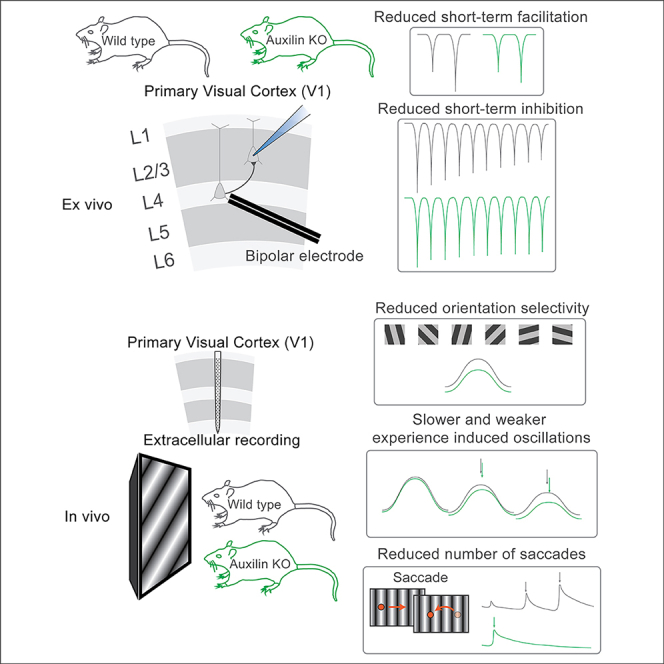

## Introduction

Synaptic transmission and plasticity play critical roles in neuronal circuit functions. A variety of proteins in the pre-synaptic terminals mediate the complex process of synaptic vesicle generation and trafficking that is essential for synaptic transmission. Auxilin is one such protein that functions as a co-chaperone for heat shock cognate 70 (Hsc70) to uncoat clathrin-coated vesicles and generates synaptic vesicles.^1^^,^^2^^,^^3^ The nascent synaptic vesicles are refilled and can then fuse with the pre-synaptic membrane for the next round of neurotransmitter release.^4^^,^^5^ Auxilin is known to function only in uncoating clathrin, mainly at the synapse and possibly at the Golgi apparatus.^6^ As expected, the clathrin-uncoating rate and vesicle endocytosis at hippocampal synapses were found to be impaired in auxilin-knockout (Aux-KO) mice.^7^ Electron microscopic studies showed that Aux-KO mice have a much higher number of clathrin cages and clathrin-coated vesicles and fewer synaptic vesicles in the pre-synaptic termini of deep cerebellar nuclei and dorsal striatum.^7^^,^^8^^,^^9^ Presumably, these defects will reduce the rapidly releasable pool (RRP) and cause functional changes pre-synaptically, though this has not yet been tested.

Deleterious mutations in *DNAJC6* encoding auxilin (*PARK19*) are associated with familial Parkinson’s disease (PD).^10^^,^^11^^,^^12^ PD patients, including *PARK19* patients, exhibit cognitive symptoms and intellectual disability, in addition to the classical motor symptoms of PD, which indicate dysfunction in cortical regions.^13^^,^^14^^,^^15^ Aux-KOs can be used to build a faithful model of *DNAJC6*-linked PD in mice and Drosophila, recapitulating the cardinal features of nigrostriatal neuronal loss, alpha-synuclein pathology, and motor deficits.^16^^,^^17^^,^^18^ As auxilin is broadly expressed in the brain and its endocytic function is universal to all synapses in the central nervous system, loss-of-auxilin is likely to impact the functions of brain regions beyond documented regions such as the hippocampus, dorsal striatum, and substantia nigra.^18^^,^^19^ Because cognitive deficits are a common symptom of PD patients, including *PARK19* patients, we utilized Aux-KO to explore the basis of these symptoms. We chose to investigate the primary visual cortex due to the ease of assays to interrogate cortical functions and as a subset of PD patients suffers from visual disturbances, such as visual hallucinations and pattern recognition deficits, attributed partially to malfunctions of the primary visual cortex in humans.^20^^,^^21^^,^^22^^,^^23^ Additionally, visual cortical functions at the synaptic level and the cortical physiology level have not been investigated in PD models, including Aux-KO mice.

Normal synaptic functions are necessary for learning. In mice, repetitive presentations of phase-reversing sinusoidal gratings over several days increase the amplitudes of stimulus-locked visually evoked potentials (VEPs) in response to the familiar stimulus.^24^^,^^25^^,^^26^ We, and others, have also shown that following continuous days of visual stimulus presentation, the theta band (4–8 Hz) oscillations are enhanced in both amplitude and duration in mice,^27^^,^^28^ which is crucial as the theta band is associated with the encoding, retention, and retrieval during learning and memory.^29^^,^^30^^,^^31^ Moreover, potentiation of theta band has also been reported as a form of experience-dependent plasticity in humans.^32^ Interestingly, previous studies have demonstrated that short-term and long-term synaptic plasticity may underlie visual experience-dependent plasticity.^33^^,^^34^ Stimulus-specific response potentiation as a form of experience-dependent plasticity is shown to induce long-term potentiation (LTP) in the mouse primary visual cortex (V1).^33^ When local manipulation of the mouse V1 prevents or reverses this LTP, it likewise prevents or reverses memory-related behavior.^25^ Altogether, these findings suggest that synaptic plasticity within a cortical circuit may underlie long-term visual memory.

Visual adaptation and mismatch responses report temporal saliency of visual stimuli, where visual cortical response reduces when the same visual stimulus is repetitively represented with short inter-stimulus intervals (adaptation response), while the response increases when an oddball stimulus is presented (mismatch response). The adaptation response has been suggested to be influenced by rapid synaptic depression,^34^^,^^35^^,^^36^ in addition to other neuronal circuit computations.^34^ Collectively, impaired synaptic vesicle endocytosis in Aux-KO mice is likely to result in deficits in visual cortical circuit functions that require synaptic plasticity. Here, we investigated how auxilin deficiency would hinder synaptic plasticity in V1, and whether visual cortical functions would be impaired both experimentally and by computational modeling.

## Results

### Attenuated paired-pulse facilitation in Aux-KO mice

**Figure 1:**
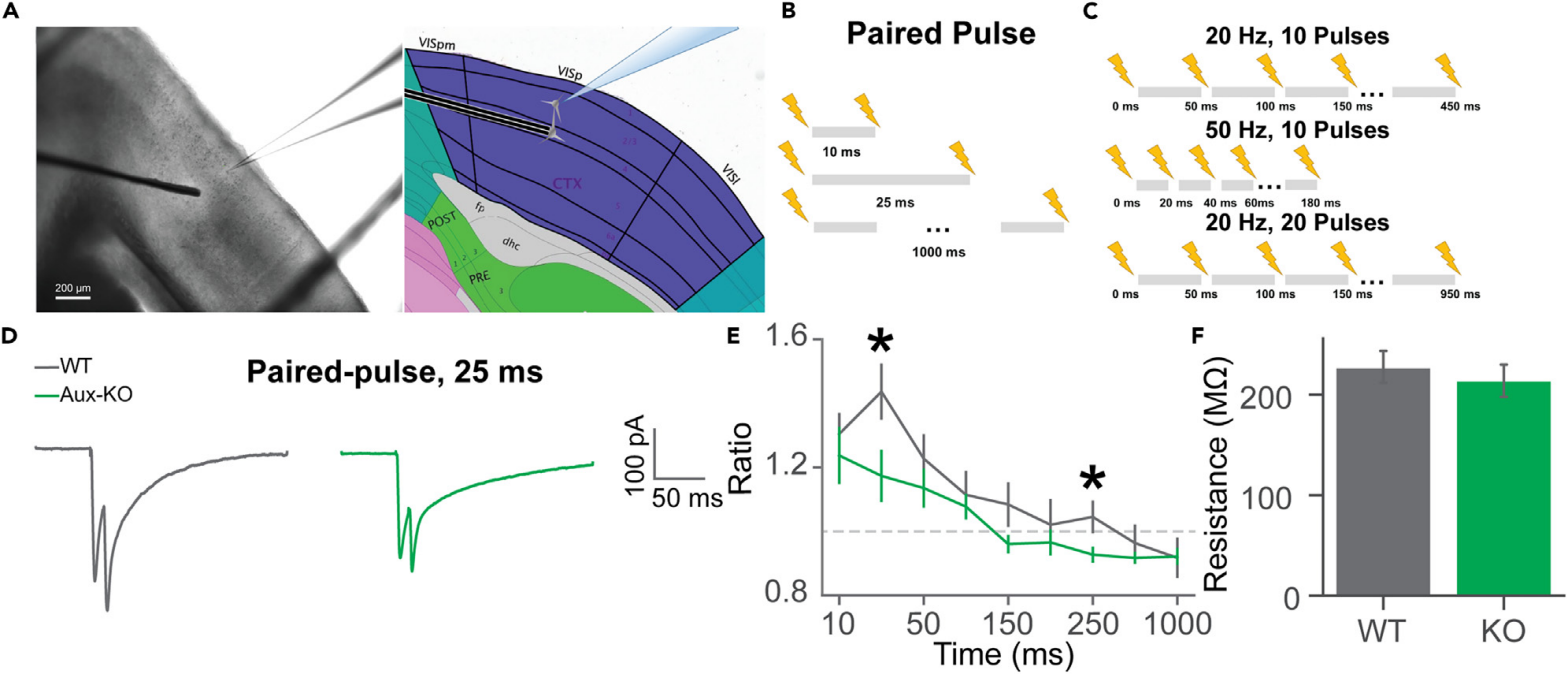
Whole-cell recordings of layer 2/3 neurons and the PPR in WT and Aux-KO mice (A) Electronic stimulation setup on the coronal brain slices with V1. The bipolar electrode was placed on layer 4 of V1, and the EPSCs of neurons in layer 2/3 were recorded by whole-cell voltage-clamp recordings held at −70mV. (B) Stimulation patterns of pair-pulse with intervals of 10 ms, 25 ms, 50 ms, 100 ms, 150 ms, 200 ms, 250 ms, 500 ms, and 1000 ms. (C) Stimulation patterns of multiple pulses by 20 Hz train of 10 stimuli, 50 Hz train of 10 stimuli, 20 Hz train of 20 stimuli. (D) Average EPSC traces when paired pulses were applied with 25 ms intervals. (E) Average PPRs of WT and Aux-KO of corresponding inter-pulse intervals as shown in (B): 10 ms (WT: N = 15 cells, Aux-KO: N = 17 cells), 25 ms (WT: N = 16 cells, p = , Aux-KO: N = 17 cells), 50 ms (WT: N = 15, Aux-KO: N = 17 cells), 100 ms (WT: N = 15 cells, Aux-KO: N = 15 cells), 150 ms (WT: N = 16 cells, Aux-KO: N = 14 cells), 200 ms (WT: N = 15 cells, Aux-KO: N = 15 cells), 250 ms (WT: N = 14 cells, Aux-KO: N = 14 cells), 500 ms (WT: N = 13 cells, Aux-KO: N = 14 cells), 1000 ms (WT: N = 12 cells, 3 mice, Aux-KO: N = 14 cells, 3 mice). Two-way ANOVA with Tukey’s post hoc. (F) Membrane resistance of neurons in WT and Aux-KO mice (WT: N = 22 cells, 3 mice, Aux-KO: N = 13 cells, 3 mice). Mann-Whitney U test. Data were presented as mean ± SEM. ∗p < 0.05.

To study how the impairment of endocytosis in Aux-KO mice affects the release of neurotransmitters and pre-synaptic short-term plasticity (STP), we performed paired-pulse stimulation, where two consecutive electrical stimulations were applied with a short interval of a millisecond to the second scale in the V1 layer 4 to layer 2/3 synapse, part of the canonical feedforward visual pathway. We performed whole-cell patch-clamp recordings of the V1 layer 2/3 pyramidal cells in acute brain slices from WT and Aux-KO mice while stimulating afferents, the neurons in layer 4 with electric current (Figure 1A). The electrical pulses evoked excitatory postsynaptic currents (EPSCs). The paired-pulse ratio (PPR) of the second to the first EPSCs allows for the characterization of the pre-synaptic properties of the synapse, namely release probability. The enhancement of the second evoked EPSC indicates a facilitation effect in the corresponding synapse, while reduction implies a depression. Previous experimental results from different synapses and simulated models report that PPR is close to 1 when the paired-pulse interval is larger than 1 s.^37^^,^^38^ We recorded responses to a range of inter-pulse intervals (IPI) in WT and Aux-KO mice (10 ms-1 s; Figure 1B). Congruent with published literature, the PPR was close to 1 for both genotypes when the ISI was 1 s (Figure 1E). The PPRs for Aux-KOs were lower than WT, with those corresponding to 25 ms and 250 ms intervals reaching significance (Figures 1D and 1E, two-way ANOVA, no significant interaction was found between genotype and IPI: F = 0.851; p = 0.559. Main effects after removing the interaction term: genotype: F = 16.56; p = 6.32E-5. IPI: F = 10.64; p = 7.45E-13. Tukey’s post hoc: 10 ms: p = 0.34; 25 ms: p = 0.0163; 50 ms: p = 0.117; 100 ms: p = 0.308; 150 ms: p = 0.0712; 200 ms: p = 0.332; 250 ms: p = 0.0289; 500 ms: p = 0.457; 1000 ms: p = 0.699). There was no difference in the membrane resistance of layer 2/3 pyramidal cells between WT and Aux-KO mice (Figure 1F, p = 0. 4932, Mann-Whitney U test). Lower PPR in Aux-KO mice indicates impaired pre-synaptic plasticity.

### Diminished short-term depression (STD) Aux-KO mice during high-frequency stimulation

**Figure 2:**
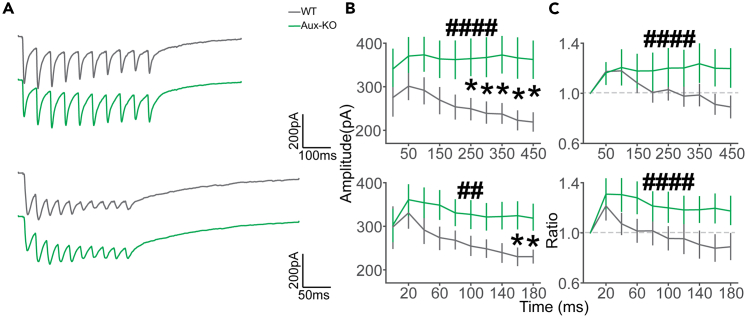
Brief 20 Hz and 50 Hz stimulations induce stronger facilitation in Aux-KO mice (A) Average EPSC traces from three stimulation strategies (top to bottom): 20 Hz stimulus train of 10 pulses and 50 Hz stimulus train of 10 pulses in WT and Aux-KO. (B) Average EPSC amplitudes upon each pulse. Top to bottom: 20 Hz train of 10 stimuli (WT: N = 12 cells, 3 mice, Aux-KO: N = 14 cells, 3 mice), and 50 Hz train of 10 stimuli (WT: N = 9 cells, 3 mice, Aux-KO: N = 13 cells, 3 mice). Two-way ANOVA with Tukey’s post hoc. (C) Average MPRs upon each pulse. Top to bottom: 20 Hz train of 10 stimuli (WT: N = 12 cells, 3 mice, Aux-KO: N = 14 cells, 3 mice), and 50 Hz train of 10 stimuli (WT: N = 9 cells, 3 mice, Aux-KO: N = 13 cells, 3 mice). Two-way ANOVA with Tukey’s post hoc. Data were presented as mean ± SEM. Two-way ANOVA: ##p < 0.01, ####p < 0.0001. Tukey’s post hoc: ∗p < 0.05.

One of the critical features of pre-synaptic machinery is the ability to function during the high-intensity conditions of high-frequency stimulation. To measure this property, we applied the 20 Hz stimulation of 10 pulses, 50 Hz stimulation of 10 pulses, and 20 Hz stimulation of 20 pulses (Figure 1C) on the direct L4 afferents while recording from L2/3 pyramidal cells. Surprisingly, overall, evoked EPSCs were significantly higher in Aux-KO mice in both 20 Hz and 50 Hz trains. Evoked EPSCs were significantly reduced in WT mice in the last few stimuli of the ten pulse-train shown by post-hoc test (Figure 2B top, two-way ANOVA, no significant interaction between genotype and time: F = 0.300, p = 0.974. Main effects after removing the interaction term: genotype: F = 39.99; p = 1.312E-9. Time: F = 0.272; p = 0.982. Tukey’s post hoc: 0 ms: p = 0.305; 50 ms: p = 0.233; 100 ms: p = 0.143; 150 ms: p = 0.0923; 200 ms: p = 0.06; 250 ms: p = 0.0356; 300 ms: p = 0.0227; 350 ms: p = 0.0145; 400 ms: p = 0.0134; 450 ms: p = 0.0104; Figure 2B bottom, two-way ANOVA, no significant interaction was found between genotype and time: F = 0.338; p = 0.961. Main effects after removing the interaction term: genotype: F = 18.63; p = 2.5E-5. Time: F = 0.827; p = 0.592. Tukey’s post hoc: 0 ms: p = 0.9; 20 ms: p = 0.594; 40ms: p = 0.217; 60 ms: p = 0.126; 80 ms: p = 0.163; 100 ms: p = 0.114; 120 ms: p = 0.105; 140 ms: p = 0.0626; 160 ms: p = 0.0341; 180 ms: p = 0.0331).

We then calculated the multiple pulse ratio (MPR = #n evoked EPSC/#1 evoked EPSC). In accordance with the paired-pulse stimulation results, we initially saw short-term facilitation during the first couple of stimuli, which evolved into a depression for WT and facilitation for Aux-KO mice by the end of the stimulus train. This difference in the ratio didn’t reach significance (Figure 2C top, two-way ANOVA, no significant interaction between genotype and time: F = 0.532, p = 0.850. Main effects after removing the interaction term: genotype: F = 8.78; p = 3.37E-3. Time: F = 0.486; p = 0.883. Tukey’s post hoc: 50 ms: p = 0.9; 100 ms: p = 0.872; 150 ms: p = 0.596; 200 ms: p = 0.340; 250 ms: p = 0.364; 300 ms: p = 0.210; 350 ms: p = 0.200; 400 ms: p = 0.118; 450 ms: p = 0.111). These results indicate that high-frequency stimulation-induced EPSCs are higher in Aux-KO mice compared to WT. 50 Hz stimulation evoked larger short-term facilitation than the 20 Hz, especially in the Aux-KO mice, and the overall trend of facilitation/depression over pulse number was similar to the 20 Hz stimulation of 10 pulses (Figure 2C bottom, two-way ANOVA, no significant interaction was found between genotype and time: F = 0.345; p = 0.958. Main effects after removing the interaction term: genotype: F = 16.88; p = 5.8E-5. Time: F = 0.987; p = 0.452. Tukey’s post hoc: 20 ms: p = 0.620; 40 ms: p = 0.216; 60 ms: p = 0.145; 80 ms: p = 0.279; 100 ms: p = 0.161; 120 ms: p = 0.218; 140 ms: p = 0.0987; 160 ms: p = 0.0786; 180 ms: p = 0.108). Overall, in the multiple pulse stimulation experiments, the ratio of consequent EPSCs to the first EPSC was higher in Aux-KO mice, which indicated lower depression effects.

### Estimation of RRP size and replenishment rate using computational models

**Figure 3:**
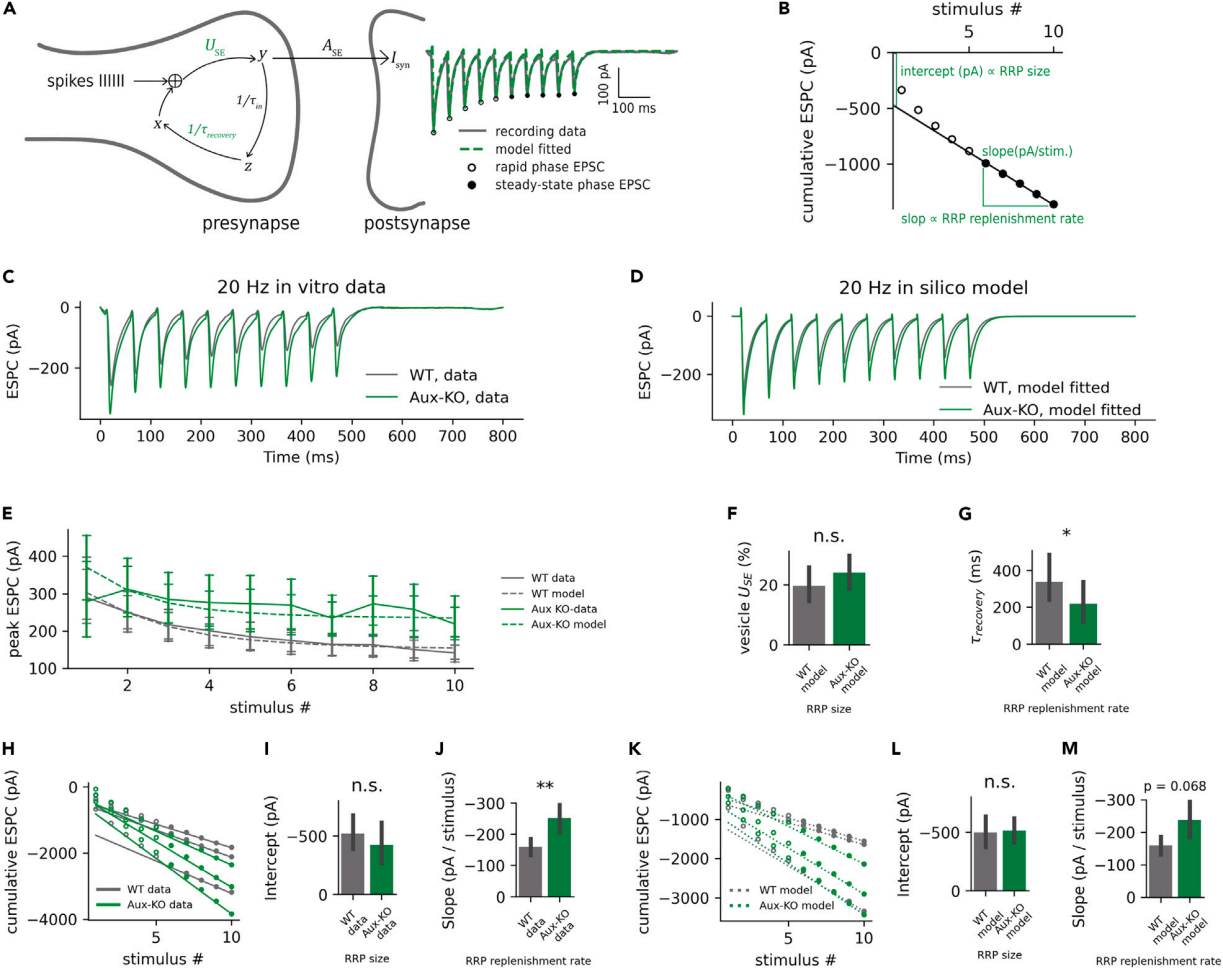
20 Hz Multiple-pulse responses in Aux-KO mice following repetitive stimulation (A) Simplified schematic of TM-model. Fitted parameters are highlighted in green color. (U_SE_: utilization of synaptic efficacy; τ_recovery_: recovery time constant; τ_in_: inactive time constant; I_syn_: postynaptic current; A_SE_: absolute synaptic strength; x/y/z: fraction of resources in recovery/active/inactive state). (B) Example for estimating RRP size and replenishment rate using SMN approach by plotting cumulative EPSCs linearly fitted at the steady-state phase. (C) Mean trace of *ex vivo* ESPC data in the WT group and Aux-KO group. (D) Mean in-silico model simulation of ESPCs under 20 Hz 10 pulses in the WT group and Aux-KO group. Raw traces were processed as described in (A). (E) Peak absolute ESPC amplitude at each stimulus pulse. The error bar indicates SEM. (F) Fitted U_SE_ from the TM model. (G) Fitted τ_recovery_ from the TM model. (H) Estimation of RRP properties in *ex vivo* ESPC data with the SMN approach. Steady-state cumulative ESPC peak amplitudes (filled circles) were linearly fitted. (I) Intercept and RRP size from SMN approach. (J) Slope and RRP replenishment rates from SMN approach. (K-M) Replication of SMN approach (F-H) in in-silico results in fitted TM-model. Data were presented as mean ± SEM. n.s. = not significant, ∗p < 0.05, ∗∗p < 0.01.

To study the mechanism of the decreased depression in multiple pulse stimulation experiments, we continued with *in silico* modeling, in which computer programs are used to create simulations of pharmacologic or physiological processes.^39^ To obtain a mechanistic understanding of the alterations of STP in Aux-KO mice, we performed computational modeling using the Tsodyks-Markram model (TM model) to fit the results of 20 Hz 10 pulses and 50 Hz 10 pulses stimulation *ex vivo* patch-clamp recording where short-term dynamics and multi-vesicular release are described (Figures 3 and S1).^40^ To better fit into the TM model, raw traces were smoothed by Savitzky-Golay filter and detrended by subtracting the upper envelope (Figures 3C–3E). We utilized an *in silico* model simulation of ESPCs under 20 Hz 10 pulses (Figures 3D and 3E). Major parameters of the TM model, vesicle utilization of synaptic efficacy (U_SE_), and recovery time constant (τ_recovery_) were fitted to the recorded ESPCs for each recorded cell (Figure 3A). The U_SE_ or RRP size was comparable between WT and Aux-KO mice according to the TM model (Figure 3F, p = 0.586, Mann-Whitney U test). τ_recovery_ was notably lower in Aux-KO than in WT mice (Figure 3G, p = 0.0162, Mann-Whitney U test). Significantly reduced τ_recovery_ in Aux-KO mice indicates a faster RRP replenishment rate. To cross-validate the major parameters from the TM model, we then estimated RRP properties in the *ex vivo* ESPC data with the Schneggenburger-Meyer-Neher (SMN) approach.^41^^,^^42^ The difference in the RRP size and RRP replenishment rate was approximated by the intercept and slope of fitted lines on steady-state cumulative ESPC peak amplitudes for each cell (Figure 3B). Steady-state cumulative ESPC peak amplitudes were linearly fitted to obtain intercept and slope, which are correlated to RRP size and RRP replenishment rate, respectively (Figure 3H). Intercept and RRP size did not significantly differ between WT and Aux-KO groups (Figure 3I, p = 0.183, Mann-Whitney U test). Slope and RRP replenishment rates were significantly increased in Aux-KO mice (Figure 3J, p = 0.005, Mann-Whitney U test). To further verify the fitting results from the TM model and SMN in *ex vivo* EPSCs, we replicated the SMN approach in our *in silico* results in the fitted TM-model, which exhibited trends in RRP properties highly close to the *ex vivo* data (Figures 3J and 3L, p = 0.644, Mann-Whitney U test; Figure 3M, p = 0.0684, Mann-Whitney U test). The model fitting results for 50 Hz 10 pulses stimulation *ex vivo* EPSCs were similar to the 50 Hz 10 pulses stimulation. No significant difference was detected in RRP size between genotypes, but the replenishment rate was higher in Aux-KO mice (Figure S1D, p = 0.639, Mann-Whitney U test; Figure S1E, p = 0.0483, Mann-Whitney U test; Figure S1G, p = 0.548, Mann-Whitney U test; Figure S1H, p = 0.006, Mann-Whitney U test; Figure S1J, p = 0.947, Mann-Whitney U test; Figure S1K, p = 0.0382, Mann-Whitney U test). While the magnitude of RRP as a potential component explaining the STP deficits in Aux-KO mice is likely to be at a similar level to WT mice, the models of observed STP showed a higher replenishment rate in Aux-KO mice.

After verifying the decreased short-term facilitation and depression in auxilin KO mice, we characterized the alterations of RRP in Aux-KO mice under sustained stimulation by the same whole-cell patch-clamp setup. We applied prolonged high-frequency stimulation with more repetitions in the same afferent neurons in layer 2/3 pyramidal cells in WT and Aux-KO mice. We repeated the 20 Hz train of 20 pulses 10–20 times with 30 s inter-train intervals. In this paradigm, it took approximately 200–400 overall pulses to nearly deplete releasable synaptic vesicles.^43^ The average evoked EPSC amplitudes over all repeats of trains in Aux-KO mice were slightly higher than the WT mice (Figure S2B, two-way ANOVA, no significant interaction detected between genotype and time: F = 0.125; p = 1.00. Main effects after removing the interaction term: genotype: F = 4.17; p = 0.042. Time: F = 1.45; p = 0.10. Tukey’s post hoc: 0 ms: p = 0.167; 50 ms: p = 0.9, 100 ms: p = 0.774,150 ms: p = 0.9, 200 ms: p = 0.765, 250 ms: p = 0.894, 300 ms: p = 0.650, 350 ms: p = 0.747, 400 ms: p = 0.606, 450 ms: p = 0.714, 500 ms: p = 0.658, 550 ms: p = 0.707, 600 ms: p = 0.654, 650 ms: p = 0.638, 700 ms: p = 0.624, 750 ms: p = 0.651, 800 ms: p = 0.609, 850 ms: p = 0.589, 900 ms: p = 0.583, 950 ms: p = 0.472). Prolonged trains tended to induce more facilitation in WT mice by two-way ANOVA, especially in the first few pulses (Figure S2C, two-way ANOVA, no significant interaction detected between genotype and time: F = 1.05; p = 0.40. Main effects after removing the interaction term: genotype: F = 35.21; p = 5.54E-09. Time: F = 4.37; p = 3.97E-09. Tukey’s post hoc: 50 ms: p = 0.001, 100 ms: p = 0.0064,150 ms: p = 0.0063, 200 ms: p = 0.0358, 250 ms: p = 0.0165, 300 ms: p = 0.0314, 350 ms: p = 0.045, 400 ms: p = 0.141, 450 ms: p = 0.274, 500 ms: p = 0.311, 550 ms: p = 0.307, 600 ms: p = 0.523, 650 ms: p = 0.684, 700 ms: p = 0.620, 750 ms: p = 0.0983, 800 ms: p = 0.668, 850 ms: p = 0.743, 900 ms: p = 0.801, 950 ms: p = 0.896). This prolonged stimulation tended to induce facilitation before the seventh pulse in the WT mice but depression in Aux-KO mice for almost every pulse (Figure S2C). While low trials of 20 Hz and 20 pulse trains resulted in facilitation mostly in Aux-KO, the high repeats of the same stimulation patterns led to depression (Figure S2C). Altogether, these data imply that the impaired pre-synaptic plasticity in Aux-KO may be attributed to increase RRP replenishment rate in brief multiple stimulations, but it attenuates during sustained repetitive neurotransmitter release events such as prolonged high-frequency stimulations according to the vesicle pool depletion hypothesis.^37^^,^^44^

### Network model linked reduced STP and STD with impaired visual-evoked response in Aux-KO mice

Before we proceeded with the *in vivo* experiment, we developed a computational model of the V1 cortex to predict the influence of the observed synaptic plasticity deficits, including STP and STD at the population level (Figure S3). This model is a spiking neural network model that simulates thousands of interconnected cortical neurons in a configuration comparable to the *in vivo* setup, as details described in the STAR Methods section. The simulation results reveal that the STP deficit can reduce the initial transient firing rates (Figure S3A), while the STD deficit affects the subsequent steady-state responses (Figure S3B). The simulated V1 responses in Aux-KO mice were attenuated and distorted under the effects of both STP and STD deficits (Figure S3C). It is possible that additional neural mechanisms, such as homeostasis and cortico-cortical feedback, may also contribute to shaping the V1 responses in Aux-KO mice. Nonetheless, this network simulation indicates that the synaptic plasticity deficits discovered in the *ex vivo* experiments are sufficient to trigger systemic changes in the temporal dynamics of the visual-evoked responses in the V1 cortex. These changes could potentially degrade the visual perception and behaviors of the Aux-KO mice in the *in vivo* experiments.

### Orientation selectivity was reduced in Aux-KO mice

**Figure 4:**
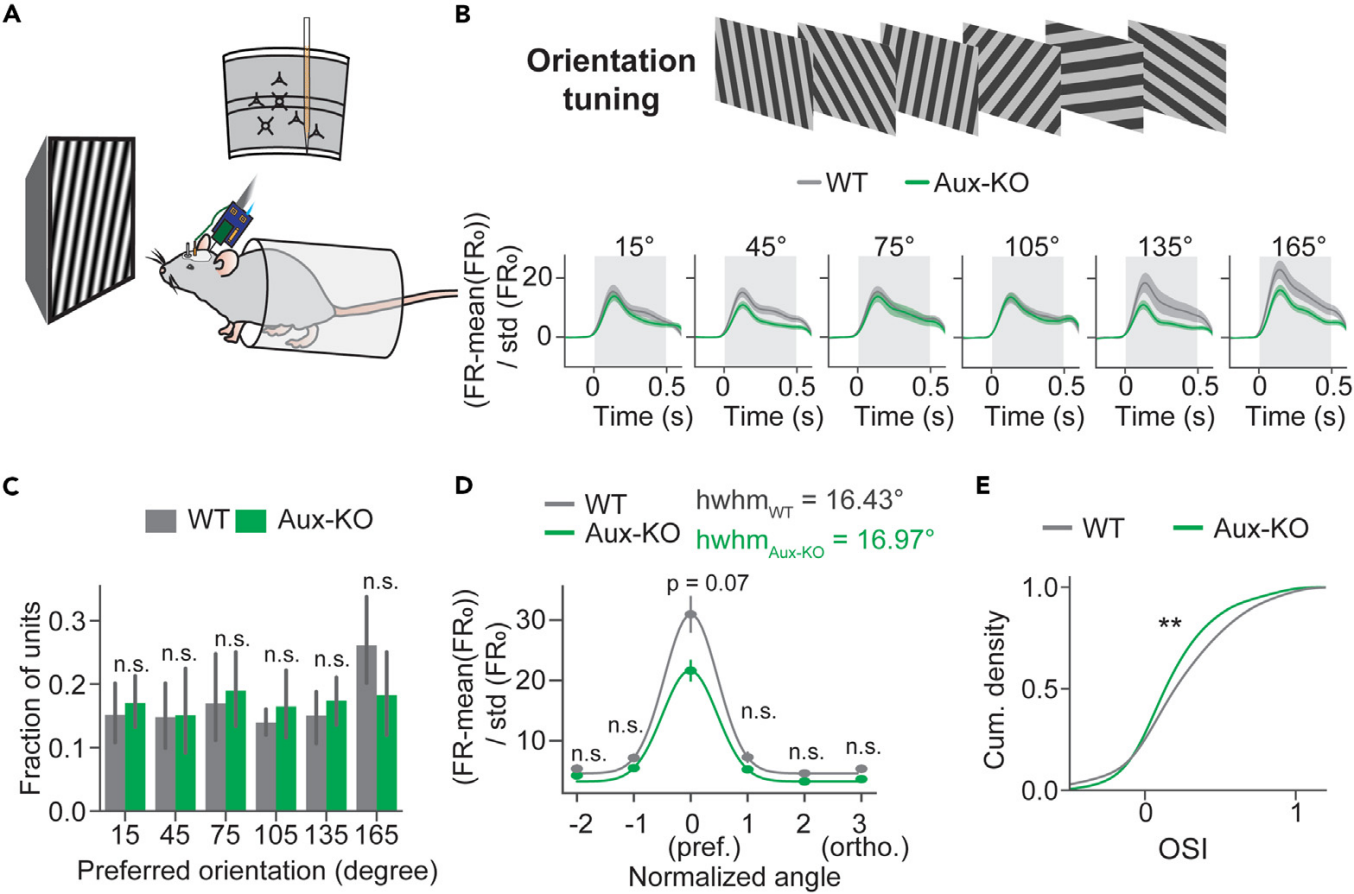
Orientation selectivity was reduced in Aux-KO mice (A) *In vivo* extracellular recordings of V1 responses were performed in head-fixed mice using 64-channel silicon probes. Visual stimulation was presented in front of the mice. (B) Static gratings with six orientations were presented to the mice. Baseline normalized response (FR-mean(FR_0_))/std(FR_0_) of all single units in response to six orientations shown below. The gray shaded area represented the visual stimulation time window. Data were presented as mean ± SEM. WT: N = 319 units, 8 mice; Aux-KO: N = 343 units, 10 mice. (C) Fractions of units’ preferred orientations are plotted in bar plots. WT: N = 8 mice; Aux-KO: N = 10 mice. The error bar represents mean ± 95% CI. Fractions of units were compared between genotypes using t-tests with FDR-BH correction. (D) Baseline normalized stimulus-locked responses averaged with the visual stimulus time window to six normalized angles plotted in a point plot. The error bar represents mean ± SEM. WT: N = 319 units, 8 mice; Aux-KO: N = 343 units, 10 mice. Units’ responses were compared between genotypes at each orientation using Mann-Whitney U tests. (E) Cumulative density plot of orientation selectivity indices (OSI). WT: N = 319 units, 8 mice; Aux-KO: N = 343 units, 10 mice. Units’ OSIs were compared using Kolmogorov–Smirnov 2 sample test.∗p < 0.05, ∗∗p < 0.01, n.s.—not significant.

**Figure 5:**
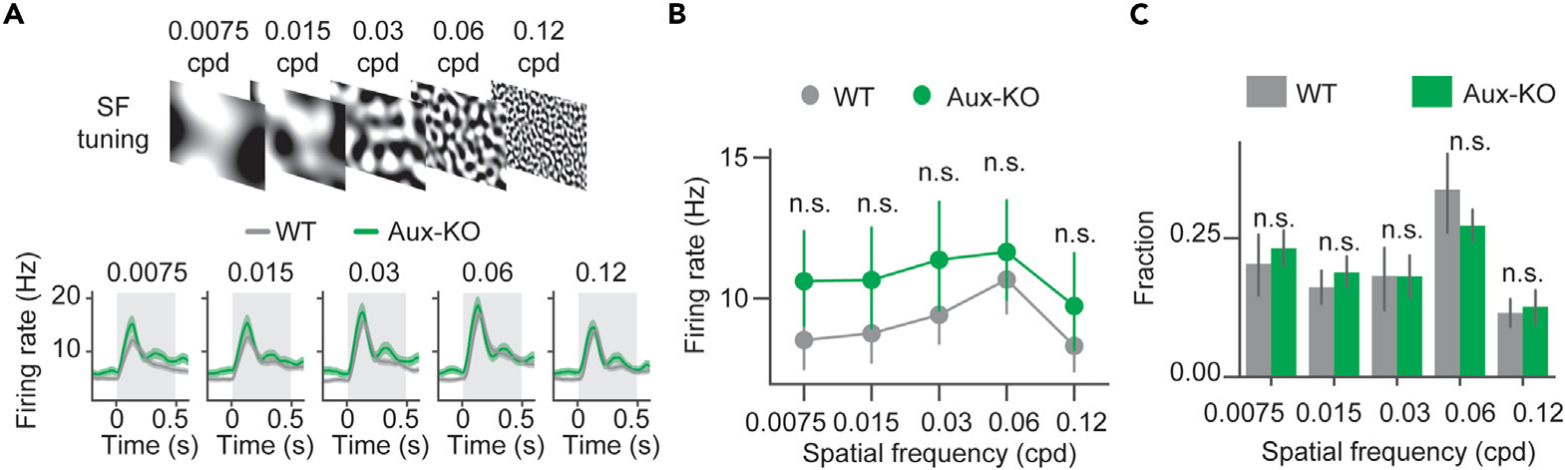
Aux-KO V1 unit population firing rates showed similar preferences for spatial frequencies as WT (A) V1 unit population firing rates (bottom) in responses to static pink noises filtered at five spatial frequencies (top). Data were presented as mean ± SEM. The gray shade represented a visual stimulation window. (B) Firing rates averaged within the visual stimulation time window in responses to five spatial frequencies. Data were presented as mean ± 95% CI. WT: N = 314 units, 5 mice; Aux-KO: N = 213 units, 4 mice. Firing rates were compared between genotypes for each spatial frequency using Mann-Whitney U tests with FDR-BH correction. (C) Fractions of units’ preferred spatial frequencies are plotted in bar plots. WT: N = 5 mice; Aux-KO: N = 4 mice. The error bar represents mean ± SEM. Fractions of units were compared between genotypes using Mann-Whitney U tests with FDR-BH correction.∗p < 0.05, ∗∗p < 0.01, n.s. not significant.

To investigate whether visual cortical functions were impaired in Aux-KO mice, we recorded extracellular activity in awake mouse V1 using a silicon probe (Figure 4A) in response to a battery of visual stimulation tests. One of the most prominent functional features of neurons in V1 is their selective responses to orientations, so we first assessed the orientation selectivity of V1 units by presenting static sinusoidal gratings of six orientations at 50% contrast to mice (Figure 4B top). The average pre-stimulus (0.2 s before the visual stimulus baseline) firing rates of all recorded units did not show significant difference between WT and Aux-KO mice (Figure S6B), suggesting no change in spontaneous V1 activity in Aux-KO mice at 3.5–5 month old. Additionally, WT and Aux-KO exhibited similar responses to static gratings at 50% contrasts (Figure S8), indicating contrast sensitivity in those mice was similar in WT and Aux-KO mice. To solely look at the response induced by the visual stimulus, we calculated baseline-normalized response ((FR-FR_0_)/std(FR_0_)) (Figure 4B bottom). The stimulus-locked response (averaged baseline-normalized response within the 0.5 s stimulus time window of the unit population) was insignificantly reduced in Aux-KO mice (Figure S6D). Then, we examined the distributions of units’ preferred orientations at which they showed the highest stimulus-locked response (Figure 4C). We found that the fractions of units’ preferred orientations were similar between both groups (Figure 4C, 15°: t = −0.529, p = 0.826; 45°: t = −0.066, p = 0.949; 75°: t = −0.409, p = 0.826, 105°: t = −0.745, p = 0.826, 135°: t = −0.758, p = 0.826, 165°: t = 1.51, p = 0.826, unpaired t tests with FDR-BH correction). Considering the heterogeneity of units’ preferred orientations, we defined the preferred orientation of each unit as angle 0 and other orientations as relative angles to the preferred orientation. We then quantified the stimulus-locked responses at the six normalized angles and found that the Aux-KO mice showed smaller response to the preferred orientation that was marginally significant compared to WT mice (Figure 4D, Normalized angle −2: U = 67097, p = 1, Orientation −1: U = 66186, p = 1, Orientation 0: U = 74750, p = 0.07, Orientation 1: U = 65266, p = 1, Orientation 2: U = 62105, p = 0.94, Orientation 3: U = 68395, p = 0.94, Mann-Whitney U test with FDR-BH correction). The half-width at half maximum (hwhm) of the Aux-KO tuning curve was slightly larger than that of the WT tuning curve (Figure 4D), indicating lower orientation selectivity in Aux-KO mice. To quantify orientation selectivity of each unit, we calculated orientation selectivity indices (OSIs) and compared the cumulative density of OSIs of WT mice and that of Aux-KO mice. The units in Aux-KO mice showed a left-shifted cumulative density curve compared to that of WT, suggesting more units in Aux-KO mice had lower OSIs than the units in WT mice (Figure 4E, statistic = 0.135, p = 0.0028, Kolmogorov-Smirnov 2 sample test). Such orientation selectivity difference was not resulted from different sensitivity to the spatial frequency between genotypes, as the V1 units’ population firing rates in responses to five static pink noises filtered at five spatial frequencies did not show significant differences (Figure 5A and 5B, 0.0075 cycles per degree (cpd): U = 37403, p = 0.105, 0.015 cpd: U = 34725.5, p = 0.541, 0.03 cpd: U = 34870, p = 0.541, 0.06 cpd: U = 34490, p = 0.541, 0.12 cpd: U = 35000, p = 0.541, Mann-Whitney U tests with FDR-BH correction). The fractions of units’ preferred spatial frequencies (the spatial frequency which the unit showed largest visually locked firing rates to) did not show significant differences between genotypes (Figure 5C, 0.0075 cpd: U = 11, p = 0.905, 0.015 cpd: U = 12, p = 0.730, 0.03 cpd: U = 12, p = 0.730, 0.06 cpd: U = 8, p = 0.730, 0.12 cpd: U = 10, p = 1, Mann–Whitney U tests). This result demonstrated that orientation selectivity was reduced in Aux-KO V1.

### Visual mismatch response was absent in Aux-KO mice

**Figure 6:**
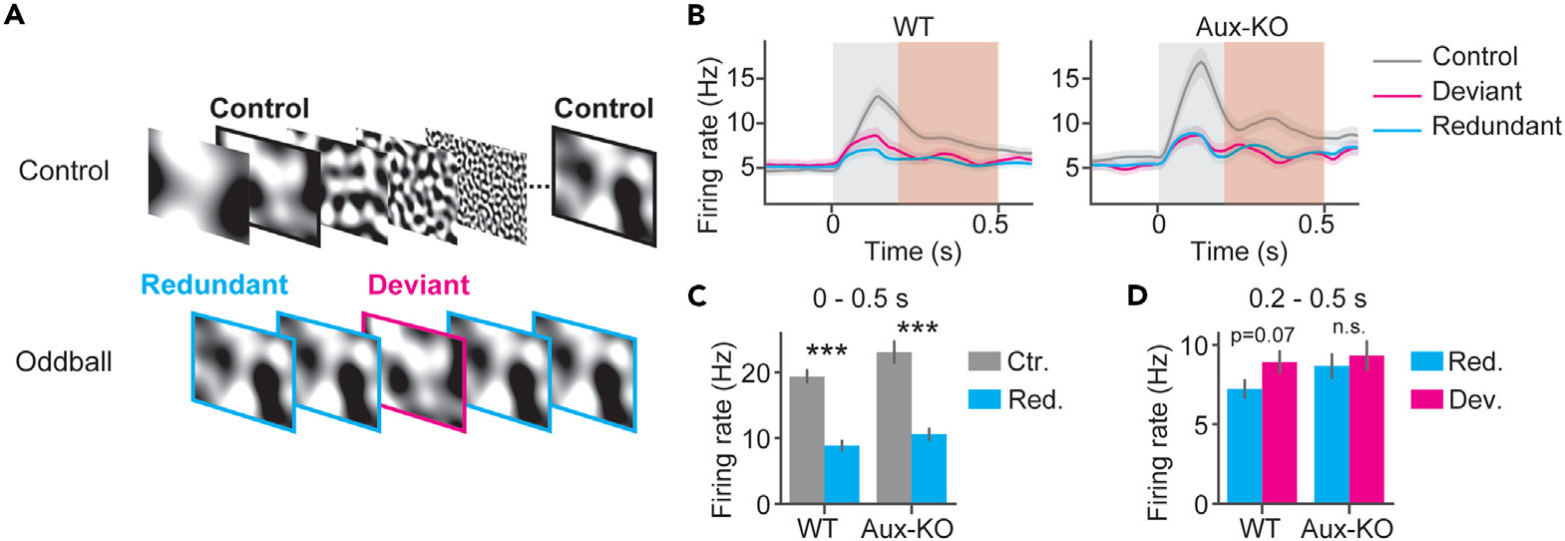
The adaptation response and mismatch response were intact in Aux-KO V1 unit population firing rates (A) Five static pink noises and their phase scrambled noises filtered at five spatial frequencies were used in a control sequence. Each visual stimulus has equal appearance frequencies. Two pink noises filtered at 0.015 cycles per degree (cpd) were used in the oddball sequences, as the redundant or the deviant stimuli. The redundant stimulus was presented in 90% of the trials, and the deviant orientation was presented in 10% of the trials. The redundant and deviant stimuli were counterbalanced in two oddball sequences. (B) V1 unit population firing rates in responses to the redundant, deviant, and control stimuli. The gray shade represented the visual stimulation time window. WT: Control: N = 323 units, 5 mice, Redundant: N = 223 units, 5 mice, Deviant: 223 units, 5 mice; Aux-KO: N = 233 units, 4 mice, Redundant: N = 188 units, 4 mice, Deviant: 188 units, 4 mice. Data were presented as mean ± SEM. (C) Units’ peak firing rates within the visual stimulation time window are plotted. Units’ firing rates were compared using Mann-Whitney U tests with FDR-BH correction. Data were presented as mean ± SEM. (D) Units’ peak firing rates within 0.2–0.5 s after the stimulus onset plotted. Units’ firing rates were compared using Mann-Whitney U tests with FDR-BH correction. Data were presented as mean ± SEM. ∗p < 0.05, ∗∗p < 0.01, n.s. not significant.

Next, we investigated sensory adaptation and visual mismatch responses using an oddball paradigm, similarly as previously established.^45^ In this paradigm, two pink noises filtered at 0.015 cpd were presented in a control sequence and an oddball sequence. The two stimuli had the same spatial frequency but different phases. In the control sequence, the two stimuli were presented together with other spatial frequencies as used in the spatial frequency tuning experiment. All stimuli were pseudo-randomly presented to the mice with equal appearance frequency (10%) in the control sequence. In the oddball sequence, one of the two pink noises filtered at 0.015 cpd was presented in 10% of trials as the deviant stimulus, and the other was presented in 90% of trials as the redundant stimulus (Figure 6A). Using unit population firing rates, the adaptation response was examined by comparing the peak firing rates within 500 ms after the visual stimulus onset, and the mismatch response was examined by comparing the peak firing rates within 200–500 ms after the visual stimulus onset (Figure 6B), similarly as previously described.^45^ Both WT and Aux-KO showed intact adaptation, as the firing rates in response to the control stimulus were significantly higher than those to the redundant stimulus (Figure 6C, WT: U = 52425, p = 1.34x10^−21^, Aux-KO: U = 29911, p = 5.75x10^−11^, Mann-Whitney U tests), yet Aux-KO did not show significant difference between the firing rates to the deviant stimulus and the firing rates to the redundant stimulus (Figure 6D, WT: U = 25532, p = 0.07, Aux-KO: U = 17557, p = 0.73, Mann-Whitney U tests), indicating absence of mismatch response. We also used orientation as stimuli in two different oddball sequences and measured mismatch response by comparing the mean potentials within 0.2–0.5 s after visual stimulation onset between responses to the deviant and redundant stimuli (Figure S9A). The responses to the deviant stimulus were significantly different from those to the redundant stimulus in WT mice but not in Aux-KO mice (Figure S9C). It was shown in rats’ EEG work that reduced mismatch response in the auditory cortex was associated with reduced theta band power,^46^ yet we did not detect significantly changed theta power in the visual cortex of Aux-KO mice (Figure S13). These results demonstrated that visual adaptation and mismatch responses were impaired in LFPs of Aux-KO mice, suggesting that the neural circuits responsible for mismatch detection were likely disrupted.

### Delayed visually evoked responses and slower visual familiarity-induced oscillations in Aux-KO mice

**Figure 7:**
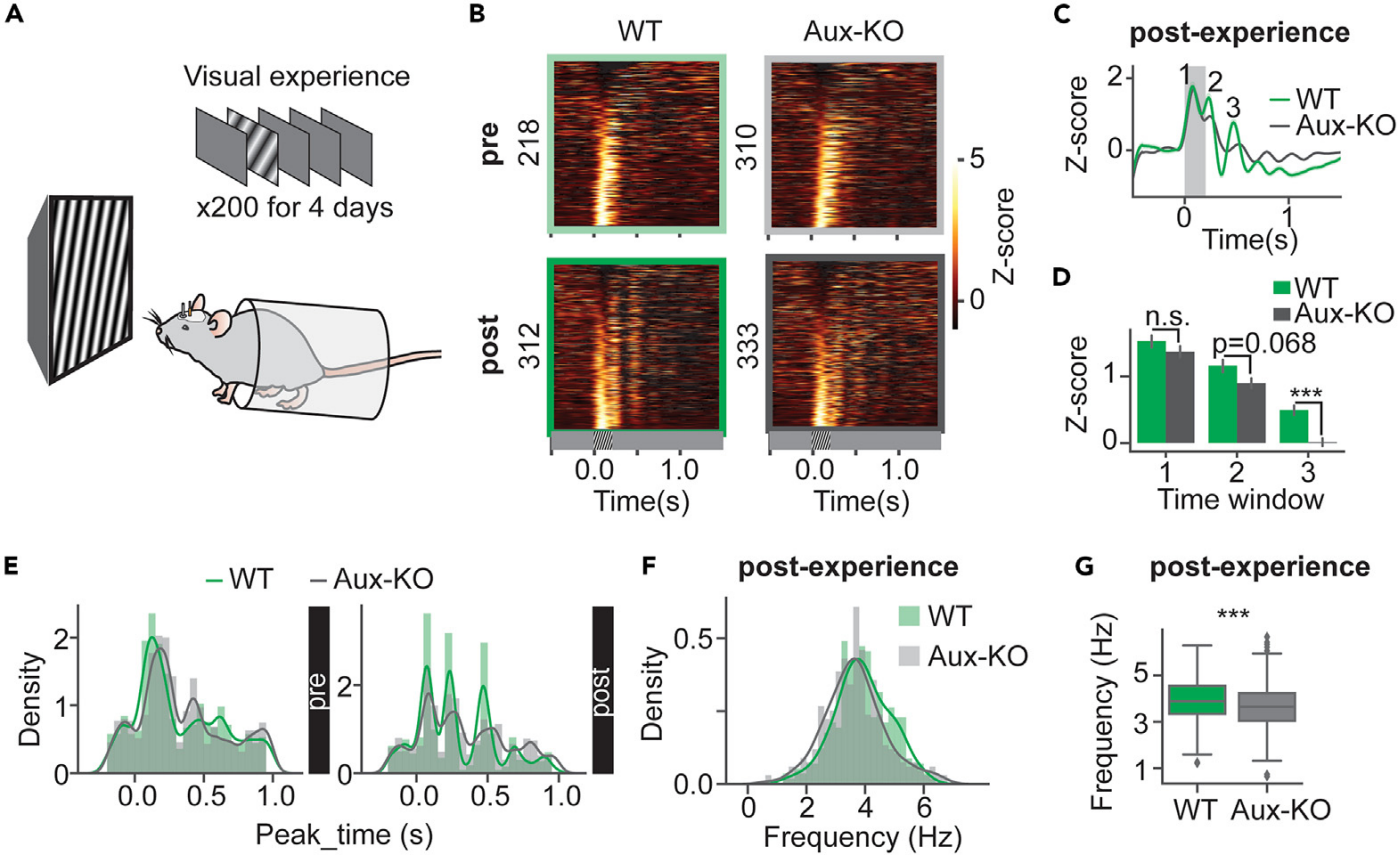
Delayed visually evoked responses and slower visual familiarity induced oscillations in Aux-KO mice (A) Visual perceptual experience in head-fixed awake mice. Drifting gratings were presented to mice over four days, with 200 presentations per day. (B) *Z*-scored firing rates of the unit population plotted in heatmaps. The numbers on the left denoted the number of units for each group. Pre: WT: N = 218 units, 7 mice, Aux-KO: N = 310 units, 10 mice. Post: WT: N = 312 units, 7 mice, Aux-KO: N = 333 units, 9 mice. (C) Unit population averaged z-scored firing rates in post-experience. The gray shaded area represented the visual stimulation time window. The numbers within the plot denoted three-time windows of response peaks (0.53–0.63s, 0.7–0.8s, 0.9–1.0s). (D) Time-window averaged z-scores in post-experience. Data were presented as mean ± SEM. Mann-Whitney U test with FDR-BH correction. WT: N = 312 units, 7 mice, Aux-KO: N = 333 units, 9 mice. (E) Response peak times are plotted in histograms. (F) Oscillation frequencies of units with more than two response peaks after experience are plotted in histograms. (G) Oscillation frequencies of units with more than two response peaks after experience plotted in a boxplot. Pre: WT: N = 184 units, Aux-KO: N = 295 units; Post: WT: N = 285 units, Aux-KO: N = 337 units. Mann-Whitney U test. ∗∗∗p < 0.001, n.s.—not significant.

We next investigated whether visual experience-dependent long-term plasticity was also impaired in Aux-KO mice using a visual perceptual experience paradigm.^28^^,^^47^^,^^48^ Awake mice were head-fixed and viewed a drifting grating stimulus (30° oriented, 0.04 cycles per degree, drifting at 2 Hz, 200 ms presentation duration, with equal luminance) for 200 repeats per day for four days. We performed 64-channel silicon probe recordings in V1 before and after the visual experience (Figure 7A).^49^ Unit firing rate z-scores were plotted in heatmaps, and the population responses showed multiple sustained peaks after the end of the familiar stimulus following visual experience in WT and Aux-KO mice (Figure 7B). These multiple peaks indicated the emergence of familiarity-evoked oscillations that we and others have described previously.^31^^,^^47^ However, in post-experience, Aux-KO mice showed smaller second and third-peak z-scores compared to those of the WT mice (Figure 7C and 6D, time window 1: U = 54979, p = 0.156, time window 2: U = 56341, p = 0.068, time window 3: U = 62868, p = 5.50x10^−6^, Mann-Whitney U test with FDR-BH correction). We next examined whether there were differences in the temporal dynamics of the familiarity-evoked oscillations between Aux-KO and WT mice. By plotting a histogram of the time of response peaks of all units (Figure 7E), we found that Aux-KO mice showed temporally delayed response peaks compared to those in WT mice in post-experience (Figure 7E right). To quantify the frequency of the familiarity-evoked oscillations, units with more than two response peaks were selected, and the oscillation frequency of each unit was quantified using the mean of inversed duration between adjacent peaks (Figure 7F). The distribution of the oscillation frequencies of units in Aux-KO mice was right-shifted compared to the distribution of WT unit oscillation frequencies (Figure 7F). The population oscillation frequency of Aux-KO mice was significantly lower compared to WT mice (Figure 7G, U = 55925, p = 4.02x10^−4^, Mann-Whitney U test), suggesting slower oscillations in Aux-KO mice after the visual experience. This result demonstrated that visual cortical neurons in Aux-KO mice exhibited delayed visual-evoked responses and slower visual familiarity-induced oscillations. Temporally delayed neural circuit activity and impaired long-term experience-dependent plasticity may underlie these impairments.

### The impaired optokinetic response in Aux-KO mice

**Figure 8:**
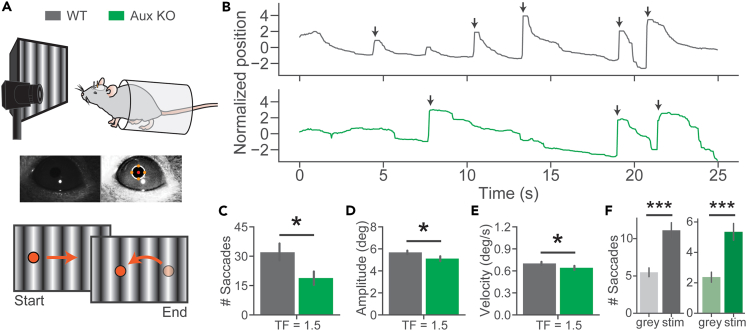
The optokinetic response is impaired in Aux KO mice, shown as reduced number of saccades and their dynamics (A) Eye movement and pupil size recordings maintained a similar head-fixation setup with the incorporation of a video camera focused on the pupil of the mouse (top). Images are representative frames from recorded videos and the coordinates outlining the pupil (middle). The visual stimulation is illustrated in the schematic below. The red dot indicated a visually tracked position. The tracked position followed the moving gratings (optokinetic response) and returned to the original position (saccade). Orange circles represent the focus point of the mouse as the visual stimulus moves across the screen throughout the recording. (B) Representative raw traces of the pupil dynamics in the x-dimension (pixels relative to the horizontal center of the video frame) across a recording session for WT and Aux-KO mice with 1.5 Hz vertical drifting gratings at 100% contrast. Arrows indicate detected saccades. (C) The total number of saccades detected during each recording session. Each mouse underwent 2 recording sessions. The numbers of detected saccades from each session were tested using the Mann-Whitney U test. WT: N = 8 sessions, Aux-KO: N = 6 sessions. (D) The average x-dimension amplitude of the detected saccades. The amplitudes of saccades were tested using the Mann-Whitney U test. WT: N = 256 saccades, 4 mice, Aux-KO: N = 113 saccades, 3 mice. (E) Maximum velocity during each saccade. The maximum velocities of saccades were tested using the Mann-Whitney U test. WT: N = 256 saccades, 4 mice, Aux-KO: N = 113 saccades, 3 mice. (F) The average number of saccades per trial during pre-trial gray screen and stimulus. Mann-Whitney U test. WT: N = 6 mice, Aux-KO: N = 5 mice. Data were presented as mean ± SEM. ∗p < 0.05. ∗∗∗p < 0.001.

Finally, to assess visually evoked behavior responses in Aux-KO mice, we measured optokinetic reflex (OKR) in Aux-KO and WT groups of mice. Awake mice were head-fixed and viewed a drifting grating stimulus (0° oriented, 0.04 cycles per degree, 1.5 Hz, 25 s presentation duration). Eye movements and pupil size changes were recorded using an infrared camera, detected, and analyzed using DeepLabCut, an open-source deep-learning toolbox for tracking user-defined features.^50^ Both WT and Aux-KO mice were able to follow the movement of the sinusoidal gratings. Rapid eye movements (saccades) were defined as the rapid shifts of eye position to capture an object in the environment (Figure 8A). Raw x-dimension movement of the pupil was centered to its mean position in the recorded field of view across the trial and showed that Aux-KO mice had less detected saccades across trials compared to the WT (Figure 8B). Further, when we grouped individual trials into complete recording sessions, we found that the Aux-KO group of mice had a significantly lower total number of saccades (Figure 8C, mean (n sessions): WT = 32.0(8), Aux-KO = 18.8(6), p = 0.046, Mann-Whitney U test). We next examined the individual saccades more specifically by first quantifying the amplitude of each. Here, we found that the WT group traveled significantly farther during each detected event (Figure 8D, mean (n saccades): WT = 5.67(256), Aux-KO = 5.11(113), p = 0.018, Mann-Whitney U test). Additionally, we investigated the maximum velocity of each event and similarly found that it was decreased in the Aux-KO group (Figure 8E, mean (n saccades): WT = 0.70(256), Aux-KO = 0.64(113), p = 0.025, Mann-Whitney U test). Finally, to confirm that the saccade detection was reliably tracking eye movement, rather than spontaneous activity, an additional experiment was performed to record mouse eye movement during a period of gray screen prior to the onset of the visual stimulus. Both WT and Aux-KO showed significant increases in number of detected saccades per trial during stimulus presentation compared to those detected during the gray screen (Figure 8F, [mean gray, mean stim, p value]: WT = [5.48, 11.11, 1.22e-6] n = 6 mice, Aux-KO = [2.37, 5.35, 1.12e-6] n = 5 mice, Mann-Whitney U test). This demonstrates that while few spontaneous eye movements may be classified as saccades, there is a significant increase in eye movement during presentation of the stimulus compared to a static gray screen in both groups, suggesting faithful eye movement tracking. Additionally, the overall pupil area of these mice showed no significant difference between periods of stimulus presentation and gray screen (Figure S15, Individual group [mean gray, mean stim, p value]: WT = [454.71, 461.06, 0.295] n = 6 mice, Aux-KO = [478.22, 484.11, 0.968] n = 5 mice. Between group p value: Gray = 0.160, Stim = 0.329, Mann-Whitney U test). Altogether, these results demonstrated impaired saccades in Aux-KO mice with slower speeds and smaller amplitudes compared to those in WT mice, suggesting potential impairments in the ON visual pathways in Aux-KO mice.

### The visual cortex of Aux-KO mice shows α-synuclein pathology at an older age

To investigate if Aux-KO mice showed alpha-synuclein pathology in the visual cortex as seen in PD patients,^51^ we first performed immunostaining of the visual cortex for pSer129-α-Syn (a marker of alpha-synuclein pathology) in young (3 months) and older mice (9 months). Aux-KO mice showed no α-synuclein pathology at 3 months of age, similar to WT (Figure S4A, 3 months, p = 0.350; Figure S5A, 3 months). At an older age of 9 months, there was a significant increase in pSer129-α-Syn expression in Aux-KO mice, suggesting alpha-synuclein pathology in the visual cortex (Figure S3A, 9 months, WT vs. Aux-KO, p = 0.0240; Figure S5A, 9 months). To determine if the visual deficits in Aux-KO can be attributed to neuronal loss, we counted neuronal nuclei immunostained for NeuN in the visual cortex. Aux-KO mice showed no neuronal loss at both 3 and 9 months of age (Figure S4B, 3 months, p = 0.725 and 9 months, p = 0.705, WT vs. Aux-KO; Figure S5B). We performed additional immunostaining for ionized calcium-binding adapter molecule 1 (Iba1), a microglial marker, and for astroglial marker glial fibrillary acidic protein (GFAP) to evaluate if there was gliosis in the visual cortex, indirect measures of neuroinflammation. At 3 months, the number of microglia and astroglia in the visual cortex of Aux-KO mice was comparable to WT (Figure S4C, 3 months, p = 0.847; D, 3 months, p = 0.752; Figure S5B). At 9 months, though there was a significant increase in astroglial number in Aux-KO mice (Figure S4D, 9 months, p = 0.0401; Figure S5B), no changes in microglial number were detected (Figure S4D, 9 months, p = 0.780; Figure S5B). These results suggest that though there is α-synuclein pathology in the older Aux-KO mice, neuron and glial numbers were preserved in the visual cortex of Aux-KO mice. Thus, impairment in visual-evoked responses and optokinetic responses seen in Aux-KO mice appears to arise from the impaired pre-synaptic plasticity and pre-synaptic endocytic perturbations in the Aux-KO mice.

## Discussion

In this study, we have performed a comprehensive characterization of the visual cortical functions in Aux-KO mice. We have demonstrated impaired synaptic short-term facilitation and depression in V1 using *ex vivo* whole-cell patch-clamp recording, impaired orientation selectivity, visual adaptation responses, experience-dependent oscillations using *in vivo* extracellular recordings, and impaired OKR responses in Aux-KO mice. We have computationally fitted our electrophysiology data to ascribe these deficits to an increased RRP replenishment rate. These results provide the first evidence for cortical synaptic plasticity and neural circuit impairments in Aux-KO mice.

### Attenuation of STP related to RRP in Aux-KO mice

Previous studies indicated the critical role of auxilin in clathrin-dependent recycling of synaptic vesicles.^5^^,^^7^^,^^52^ In the *ex vivo* single paired-pulse stimulus experiment, the WT showed higher PPR than the Aux-KO. Both facilitation and depression effects are more pronounced in WT than Aux-KO mice and are consistent with the facilitation-depression model (FD model) to account for the use-dependent changes in transmitter release.^53^ In *ex vivo* multiple-pulse short-term stimulation experiments, however, Aux-KO synapses had an overall higher MPR probably due to smaller depression effects since synapses with lower depression effects also had higher MPR in the FD model.^53^ Therefore, we explored which components of the pre-synaptic release machinery were affected in Aux-KO mice by SMN approach^41^ and discovered upregulated RRP replenishment rates, while RRP size was indistinguishable in Aux-KO compared to WT. The SMN approach is based on certain assumptions and comes with inevitable pitfalls.^54^^,^^55^ The RRP size and replenishment rates were also supported by the TM model, verifying the robustness of the SMN approach. Previous studies showed that the function of auxilin in synaptic vesicle recycling was partially compensated by the over upregulation of GAK in Aux-KO mice as much as 3 fold.^7^^,^^56^ The upregulated RRP replenishment rates in the brief repetitive stimulations of 20–50 Hz may be explained by the functional overcompensation of GAK. Interestingly, prolonged stimulus trains would be expected to lead to an exhaustion of the neurotransmitter stores leading to depression. And indeed, during the prolonged stimulus trains, Aux-KO mice exhibited depression which can be attributed to the stronger depletion of RRP.^37^^,^^57^ In most synapses between PCs, STD during repetitive stimulation follows the 1/f rule which is the synaptic response amplitude decreases in inverse proportion to the pre-synaptic firing frequency at the steady state.^58^^,^^59^^,^^60^ However, increases in the pre-synaptic firing rate above a certain limiting frequency do not increase in post-synaptic firing proportionally,^58^^,^^59^ which aligned with what we found that the baseline population firing rate is similar between WT and Aux-KO mice when the pre-synaptic plasticity is impaired in Aux-KO mice. Our network model also validated the *in silico*-simulated baseline of normalized firing rates is comparable between WT and Aux-KO mice (Figure S3). Consistent with our findings, decreased paired-pulse depression in the basal ganglia has been described in dystonia and PD patients.^61^ The authors found that the paired-pulse depression could be restored after high-frequency stimulations *in vivo*, consistent with our findings of depression observed in Aux-KO mice during the prolonged stimulus trains. Although our experiments were conducted on acute brain slices and the synapses being investigated are different, our results showed a similar trend of alteration of the pre-synaptic plasticity in Aux-KO mice. This implies that parkinsonism may share similarities in the synaptic function deteriorations in the cortical, brainstem, and spinal levels.^61^^,^^62^ In addition to the PD-like motor deficits, dopamine dysregulation, and alpha-synuclein pathology, Aux-KO mice may demonstrate some of the pathophysiology of PD at the synaptic level.^18^ Overall, our findings suggest that neural network homeostasis was disrupted due to attenuated STP in the Aux-KO mice since replenishment of the RRP is partially dependent on the endocytic recycling of synaptic vesicle membranes.^63^^,^^64^

### Impaired visual responses in V1 of Aux-KO mice

In our assessment of visual cortical response *in vivo*, we found reduced orientation selectivity with similar visual acuity, absence of mismatch responses, and slower visual experience dependent oscillations in Aux-KO mice. Recent work revealed lower proportion of functional vesicles for neurotransmitter filling in young Aux-KO mice at 3 months old,^65^ suggesting potential impairments in synaptic functions. Here, we first demonstrated reduced short-term potentiation and reduced short-term depression in the *in vitro* work. Such synaptic deficits in the visual system likely alter circuit development, neural network activity, and subsequent visual behaviors. Indeed, we found that orientation selectivity, as a feature of visual cortical neurons that is dependent on circuit computation, was decreased in Aux-KO mice, compared to WT mice. The decreased selectivity was not attributed to decreased spontaneous activity or decreased contrast sensitivity in auxilin-KO mice. Lower visual acuity, such as lower spatial resolution, could contribute to the decreased orientation selectivity,^66^ yet the visual acuity was found comparable to WT, as indicated by comparable responses to five spatial frequencies in Aux-KO mice as in WT. This decreased orientation selectivity could be a reflection of disrupted circuit functions during development, such as misalignment of orientation preference when receiving inputs from the ipsilateral or contralateral eye during the critical period.^67^ Short-term synaptic plasticity and local inhibition could potentially also contribute to sensory adaptation response and mismatch responses, respectively.^34^^,^^68^ To probe whether there were deficits in these responses in Aux-KO mice, we used an oddball paradigm that was similar to what we previously developed.^45^ In Aux-KO mice, the visual adaptation response was present in unit population firing rates and LFPs, while the mismatch response was absent in either unit population firing rates or LFPs. The lack of mismatch responses in Aux-KO mice could potentially attributed to deficits in local inhibitory connections.^68^^,^^69^

The unit population responses in Aux-KO mice exhibited delayed oscillation peaks, and slower oscillation frequencies, compared to the oscillations in WT. Such altered cortical oscillations were also reported in parkinsonian patients in different contexts,^70^^,^^71^ suggesting temporally altered circuit activity. The shifted frequency of the familiarity-evoked oscillations in Aux-KO mice is similar to the previously described frequency shift in Fmr1 KO mice, which was accompanied by reduced functional connectivity between excitatory neurons and inhibitory neurons.^28^ This oscillation frequency shift seen in Aux-KO mice could likely be caused by the changes in the excitatory/inhibitory (E/I) balance reported in several neurodegenerative diseases, including Parkinson’s disease.^72^ Further in-depth investigation of how synaptic plasticity leads to E/I balance impairment and oscillatory dysfunction in PD requires future studies.

### Deficits of optokinetic response in Aux-KO mice

Studies reveal that locomotion and ambulatory behavioral dysfunction in Aux-KO mice were age-dependent and progressive.^73^ Nevertheless, how visual motor functions are compromised in Aux-KO mice is yet to be determined. The generation of saccades, both visually guided and voluntary, is controlled distinctly by parallel descending pathways involving the cerebral cortex (the frontal and parietal areas), basal ganglia, the superior colliculus, and the brainstem saccade generator.^74^ Therefore, eye movement, especially in the saccadic system, is an indicator of the integrity of the network comprising cortical (mainly frontal and parietal) and subcortical areas.^75^ In another familial form of PD (PARK9 or Kufor-Rakeb syndrome, #606693), caused by mutations in ATP13A2 (PARK9) encoding a lysosomal P-type ATPase,^76^ patients displayed typical eye movement abnormalities, including slow saccades with worse precision and increased latency of horizontal saccades.^77^^,^^78^ Additionally, sporadic PD patients have also been documented to show abnormal saccades.^79^^,^^80^ We recorded the visually induced saccades in Aux-KO mice and found a decreased number of saccades and impaired dynamics. This impaired optokinetic response may reflect the neural circuitry dysfunction that we observed by *ex vivo* and *in vivo* electrophysiology approaches and could be a hallmark for neural functional disturbance in prodromal PD. Our findings warrant investigating optokinetic responses in other endocytic mutants linked to PD, such as synaptojanin 1 (PARK20) and RME-8 (PARK 21).

### α-Synuclein pathology in older Aux-KO mice

Aux-KO mice were previously shown to develop cardinal features of PD, including accumulation of pSer129-α-Syn in the nigral dopaminergic neurons, which was accompanied by gliosis and neuronal loss in the nigra.^73^ The presence of similar pSer129-α-Syn accumulation even in the visual cortex at later ages suggests Aux-KO mice develop cortical α-synuclein pathology as seen in the late stages of PD.^81^^,^^82^ Visual hallucinations were strongly correlated with cortical α-synuclein pathology (Harding et al., 2002). Interestingly, alpha-synuclein pathology was variable within Aux-KO mice, similar to what is seen in the visual cortex of PD patients.^51^ The absence of cortical neuronal loss in the Aux-KOs was in line with what is seen in PD patients.^83^ Overall, these pathological changes are intriguing as they appear only at later ages, suggesting that the impairment in visually evoked and optokinetic responses seen in younger Aux-KO mice may represent early pathophysiological events. This may also be attributed to the previously seen synaptic endocytosis and neurotransmitter compartmentalization dysfunctions in relatively young Aux-KO mice in other brain regions.^7^^,^^73^

Taken together, our findings demonstrated the abnormal STP in the synapses of the visual cortical circuits in Aux-KO, as well as impaired visual cortical responses. Evidence from animal and human studies suggests that different synapses in the central nervous system have distinct alterations in the strength and dynamics of plasticity in parkinsonism.^84^^,^^85^^,^^86^^,^^87^^,^^88^ The mechanism of visual deficits in PD has been less studied despite the fact that up to 40% of the patients with PD suffered visual hallucinations,^23^ which were indicated as a clinical sign of differential diagnosis of parkinsonism.^89^ STP, as well as LTP, plays critical roles in information processing and memory formation.^90^^,^^91^ The aberrant STP and the potentially disrupted excitation/inhibition balance due to the impaired strengths of excitatory and inhibitory synapses could also be critically affected in PD.^92^^,^^93^ We have demonstrated such short-term synaptic plasticity deficits together with a lack of visual adaptation responses *in vivo*. Additionally, weaker and slower experience-dependent oscillations in Aux-KO mice are consistent with the impairments in long-term plasticity that are typically associated with learning and memory, suggesting similar mechanisms might lead to cognitive dysfunction in patients carrying auxilin mutations.

### Limitations of the study

Aux-KO mice demonstrate PD-like behavioral abnormalities after 6 months old. However, these behavioral impairments were not detected in Aux-KO mice at 3 months old.^9^ Our quantifications of α-synuclein pathology are consistent with these reports. Our *in vitro* and *in vivo* experiments were conducted in 3-5-month-old mice. Thus, future studies of synaptic and neural circuit mechanisms underlying the onset of behavioral aberrations will be informative on older Aux-KO mice. Both short-term and long-term synaptic plasticity may represent the underlying cellular mechanisms of visual experience-dependent plasticity,^33^^,^^34^ but our work addresses only the pre-synaptic short-term plasticity impairments, leaving the research of long-term synaptic plasticity for the future. Finally, some studies have reported decreased inhibition in the motor cortex of patients with PD.^94^^,^^95^^,^^96^^,^^97^ Mismatch responses can potentially be explained by local inhibition,^98^ which represents another attractive avenue of research into the pathophysiology of auxilin dysfunctions at the neural circuits and systems level.

## STAR★Methods

### Key resources table

**Table:** 

REAGENT or RESOURCE	SOURCE	IDENTIFIER
**Chemicals**		

Monosodium Phosphate	Santa Cruz	sc-202342
Sodium Bicarbonate	DOT scientific, Inc	DSS22060
Dextrose	DOT scientific, Inc	7203B
Sodium Chloride	DOT scientific, Inc	DSS23020
Calcium Chloride	DOT scientific, Inc	DSC20010
Magnesium Chloride	DOT scientific, Inc	DSM24000
Potassium Chloride	DOT scientific, Inc	DSP41000
NMDG	Sigma-Aldrich	M2004
Ascorbic Acid	DOT scientific, Inc	DSA50040
Thiourea	Acros Organics	CAS 62-56-6
Na-pyruvate	Sigma-Aldrich	P2256
Phosphocreatine	Sigma-Aldrich	P7936
GTP	Sigma-Aldrich	G8877
ATP	Sigma-Aldrich	A9187
HEPES	Sigma-Aldrich	H3375
QX314- Cl	Sigma-Aldrich	L1163
TEA-Cl	Sigma-Aldrich	T2265

**Experimental models: Organisms/strains**		

C57BL/6	The Jackson Laboratory	C57BL/6
Auxilin KO	NIH	Auxilin KO

**Software and algorithms**		

Python	Python	Version 3.9.7
Anaconda Distribution	Anaconda Inc.	Anaconda for Python 3
Matlab	Mathworks	Matlab
SAS	SAS Institute	SAS

**Other**		

C&B-Metabond® Quick! Cement System	Parkell	S380
ZIP Kicker CA accelerator	ZAP Glue	#PT-27
LOCTITE super glue ultragel control	LOCTITE	SKU #688626 (Home Depot)
Animal Temperature Controller	World Precision Instruments (WPI)	ATC-2000
Motorized Stereotax	Neurostar	Single robot stereotax
Isoflurane Vaporizer	Parkland Scientific	V3000PK
Micromanipulator	Scientifica	PatchStar Micromanipulator with PatchPad
Stereo Microscope	AmScope	SM-4TZ-144A
Aluminum Breadboard 12" x 18" x 1/2", 1/4"-20 Taps	Thorlabs	MB1218
Miniature V-Clamp, 0.42" Long, M4 Tapped Hole	Thorlabs	VH1/M
Ø1.25" Studded Pedestal Base Adapter, 1/4"-20 Thread	Thorlabs	BE1
Short Clamping Fork, 1.24" Counterbored Slot, 1/4"-20 Captive Screw	Thorlabs	CF125C
Swivel Post Clamp, 360° Continuously Adjustable	Thorlabs	SWC
Ø1/2" Optical Post, SS, 8-32 Setscrew, 1/4"-20 Tap, L = 6"	Thorlabs	TR6
Ø1/2" Optical Post, SS, 8-32 Setscrew, 1/4"-20 Tap, L = 3"	Thorlabs	TR3
Large V-Clamp with PM4 Clamping Arm, 2.5" Long	Thorlabs	VC3
Ø1" Pedestal Pillar Post, 1/4"-20 Taps, L = 3"	Thorlabs	RS3P
Adapter with External 8-32 Threads and External 1/4"-20 Threads	Thorlabs	AP8E25E
Rotary encoder	U.S. Digital	H5 ball bearing optical shaft encoder
Acrylic disk, 6” diameter, 1/8” thick	Amazon	Acrylic disk, 6” diameter, 1/8” thick
64 Channel Silicon Probe	Masmanidis Lab, UCLA	64D
Acquisition Board	OpenEphys	Acquisition Board
128 Channel Amplifier Board	Intan Technologies	RHD2000
Arduino Board	Arduino	A000066
I/O Board	OpenEphys	I/O Board
Electroplating Board	Intan Technologies	RHD2000 Electroplating Board
Interface Cable	Intan Technologies	RHD2000 SPI interface Cable
Camera	Thorlabs	DCC1545M
Camera Lens	Thorlabs	MVL50M23
Infrared Illuminator	Towallmark	Towallmark Crazy Cart 48-LED CCTV Ir Infrared Night Vision Illuminator
Patch Clamp Digitizer	Molecular Devices	Multiclamp 700B
Patch Clamp Amplifier	Molecular Devices	Digidata 1550A
Patch Software Suite	Molecular Devices	pCLAMP 10
Capillary Glass Micropipette	Sutter Instruments	BF150-86-10
Micropipette Puller	Sutter Instruments	P-97
Vibratome	Leica	VT1000
bipolar electrode	FHC	CE2C55
High Current Stimulus Isolator	World Precision Instruments (WPI)	A365

### Resource availability

#### Lead contact

Further information and requests for resources and reagents should be directed to and will be fulfilled by the lead contact, Alexander A. Chubykin (chubykin@purdue.edu).

#### Materials availability

This study did not generate new unique reagents.

### Experimental model and study participant details

#### Animals

All experiments on the mice have been approved by the Purdue Animal Care and Use Committee (PACUC). The male and female Aux-KO mice and C57BL/6J, wild-type mice, were kindly provided by Dr. Lois E. Greene from NIH. Aux-KO mice were previously described^7^ and were bred to C57BL6/J mice to have the same genetic background.^18^ All the mice were subject to the standard circadian cycle of twelve-hour light and twelve-hour dark. The genotypes were blinded to the experimenter during the data collection. *In vivo* extracellular recordings were performed on a group of 3.5-5 month-old mice, an age where Aux-KO do not exhibit motor symptoms. *Ex vivo* slice patch clamp recordings were performed on the 4.5-month-old mice after *in vivo* recordings.

### Method details

#### Acute V1 slices preparation

The mice were anesthetized with a mix of saline diluted ketamine (10mg/ml), and xylazine (16mg/ml). The dosage is 0.01 ml/g and deep anesthesia was confirmed by toe pinching. The perfusion and acute slice recovery strategies were the same as described in the paper of Ting et al.^99^ The mice were transcardially perfused with room temperature NMDG artificial cerebrospinal fluid (NMDG ACSF, composition in mM: 92 NMDG, 30 NaHCO_3_, 20 HEPES, 10 dextrose, 10 MgCl_2_, 5 ascorbic acid, 3 Na-pyruvate, 2.5 KCl, 2 Thiourea, 0.5 CaCl_2_, 1.25 NaH_2_PO_4_; pH=7.3-7.4) till livers turned pale. The mice were then decapitated, and the brains were extracted and trimmed to be glued to the cutting chamber of the vibratome (VT 1000 Leica). 300-micrometer coronal brain sections containing the V1 were cut in ice-cold NMDG ACSF by vibratome with continued carbogenation (carbogen: 95% O_2,_ and 5% CO_2_). Following the protocol of Ting et al.,^99^ we incubated the brain slices from 5-month-old mice in 32°C NMDG ACSF for seven to eight minutes. The slices were then quickly transferred to an incubation chamber filled with room temperature HEPES holding ACSF (composition in mM: 92 NaCl, 30 NaHCO3, 25 dextrose, 20 HEPES, 5 ascorbic acid, 3 Na-pyruvate, 2.5 KCl, 2 thiourea, 1.25 NaH_2_PO_4_, 1 CaCl_2_, 0.8 MgCl_2_; pH=7.3-7.4). After equilibrium in HEPES holding ACSF bubbled with carbogen for at least one hour, the slices were ready for clamp-patch recording, and the slices could be kept healthy for 10 h in the incubation chambers.

#### Electrophysiology and data acquisition for whole-cell patch-clamp recording

Patch-clamp recordings were conducted on an electrophysiology rig (SliceScope Pro 1000, Scientifica).^28^ The brain slices were settled down in a submersion-type recording chamber with a slice hold down (Warner Instrument) and perfused with carbogenated ACSF (composition in mM: 124 NaCl, 26 NaHCO_3_, 10 Dextrose, 2.5 KCl, 2 CaCl_2_, 1.25 NaH_2_PO_4_, 0.8 MgCl_2_; pH=7.3-7.4) continuously during the whole recordings. Neurons were imaged with an infrared differential interference contrast optics from the rig equipped with a 4x objective lens and a 40x water immersion objective lens (Olympus) and ROLERA™ Bolt Scientific CMOS Camera (qImaging). The resistance of the recording pipette was 4-7 MO, and the intracellular pipette solution (composition in mM: 103 D-gluconic acid, 103 CsOH, 20 HEPES, 10 Na_2_- phosphocreatine, 5 QX314- Cl, 5 TEA-Cl, 4 Mg-ATP, 2.8 NaCl, 0.3 Na_2_-GTP, 0.2 EGTA; pH=7.2-7.3). QX314 and cesium in the internal solution block the firing of action potential in patched cells. All recordings were conducted with voltage-clamp mode with a holding potential of -70mV. The position of the V1 layer 4 and layer 2/3 areas was identified under a 4x objective lens. As shown in Figure 1A, the bipolar electrode (diameter: 25 μm, FHC CE2C55) was placed in the V1 layer 4 and the pyramidal cells recorded were in the L2/3 and right at the axon tracts of the neurons stimulated by the electrode. The intensity of the output current at which the evoked EPSCs are 40% to 60% of the maximal values was used. The stimulation current was generated by the constant current stimulus isolator (WPI A365). Electrophysiological data were acquired with an amplifier (Multiclamp 700B, Molecular Devices) and a digitizer (Digitata, 1550, Molecular Devices). The acquired data were recorded with Clampex 10.4 (Molecular Devices) and filtered with a 20k Hz low pass filter. The evoked excitatory postsynaptic currents (EPSC) were recorded in a whole-cell voltage patch-clamp, holding at -70mV. The membrane resistance of each patched neuron was recorded from the readout of the Clampex 10.4. The stimulus was applied by the temporal pattern indicated in Figures 1B and 1C. The duration of a current stimulation was 0.2 ms. Four to six V1 L2/3 pyramidal neurons were recorded in each mouse, and each stimulation protocol was repeated three to five times for all cells as a trial repeat except for the prolonged training protocol, which was repeated fifteen to twenty times for each neuron. The interval between the trails is 10 sec for pair-pulse simulations, and 30 sec for multiple-pulse stimulations.

#### Head-post implantation surgery

The mouse was first anesthetized using 5% isoflurane in oxygen or room air (SomnoSuite system) in an induction chamber. After deep anesthesia was confirmed (no reflex after a foot pinch), the mouse was transferred to a stereotaxic frame (Kopf or NeuroStar) and maintained anesthetized using 1.5-2% isoflurane delivered through a nose cone while the body temperature was monitored and kept at 37°C. Eye ointment was applied to prevent dryness. The skin over the mouse skull was removed using sterile scissors, and 3% hydrogen peroxide was applied to the skull to remove connective tissues. V1 was labeled using a permanent marker using stereotaxic coordinates (relative to lambda: V1: ±3.0 mm lateral, 0.8 mm anterior). A nail (headpost) was adhered to the mouse skull using superglue at 0.7 mm anterior to the bregma, and a gold-plated reference pin (WPI 5482) was inserted through the skull and above the brain surface (0.5 mm anterior to the bregma). The skull, the reference pin, and the headpost were covered with Metabond (Parkell S380) in the end. Mice were allowed for 3 days to recover before experiments.

#### Head-fixation setup and visual stimulation

After the mice recovered from the surgery, we let the mice habituate to the head-fixation setup first. During the habituation, the mouse was head-fixed by the headpost and was loosely restrained by a customized tube. A visual stimulation monitor was placed at 17 cm in front of the mouse. While the mouse was head-fixed, a gray screen (mean luminance 73 cd/m^2^) was shown to the mouse for 1.5 hours per day for at least 4 days before electrophysiology recordings. Extracellular recordings were taken after mice were habituated to the setup.

Visual stimuli were generated and displayed using PsychoPy. For the visual adaptation and mismatch negativity experiment using gratings, two static gratings (50% contrast, 0.04 cycles per degree, 45° or 135° counter-clockwise to the vertical orientation, with equal luminance as the pre-stimulus gray screen, with 0.5 s presentation duration) of 90-degree difference in their orientations were used as either deviant or redundant stimuli. In 300 presentations of visual stimuli, the deviant stimulus was presented in 10% of trials (with linearly increased appearance probability in the sequence), and the redundant stimulus was presented in the other 90% of trials. To counterbalance potential preferences for the two stimuli, two sequences were presented, with one sequence using 45° as the redundant stimulus and the other sequence using 135° as the redundant stimulus. An additional control sequence with equal appearance probability was presented at the beginning of the recording, where static gratings at six orientations (including the two selected for measurements of visual adaptation and mismatch negativity) were pseudorandomly presented, with 50 presentations for each stimulus. In all sequences, each stimulus was presented for 0.5 s, separated by a variable 1.2-1.8 s interval in between presentations. For the visual adaptation and mismatch experiment using filtered pink noises, a pink noise was first generated in Matlab. The pink noise and its phase-scrambled image were filtered at 0.015 cpd for the oddball sequence, and filtered at four more spatial frequencies for four additional spatial frequencies. For the contrast detection experiment, the 45° and 135° static gratings were pseudorandomly presented to mice at 5 contrasts (10%, 30%, 50%, 80%, and 100%, with 0.2 s presentation duration) with 20 trials at each contrast.

For the visual perceptual experience, drifting gratings (oriented 30 degrees counter-clockwise away from the vertical direction, 0.04 cycles/degree, 2 Hz, with equal luminance as the pre-stimulus gray screen, with 0.2 s presentation duration) were presented to the mouse for 200 trials per day for four days. Extracellular activities in V1 were recorded before and after the visual perceptual experience when the same stimulus was presented for 20 trials.

For the optokinetic response experiment, a vertically drifting gratings (100% contrast, 0.04 cycles per degree, 1.5 Hz, with 20 s presentation duration) was presented to mice. In a separate, additional experiment, a matching visual stimulus was shown with a static grey screen shown at the start and end of each trial recording.

#### Extracellular recording preparation and data acquisition for extracellular recording

After habituation or visual experience, extracellular activities in V1 were recorded using a 64-channel silicon probe in head-fixed mice. The 64-channel silicon probe (64D) has three columns of channels with 25 μm vertical spacing between adjacent channels, and 20 μm horizontal spacing between adjacent channels.^49^ The skull over the pre-labeled V1 position was removed when the mouse was anesthetized on the stereotaxic frame, and then the mouse was transferred to the head-fixation setup. The probe was first positioned above the craniotomy and then was inserted into the brain perpendicularly to the brain surface using a Scientifica manipulator. Sterile ACSF was added on top of the brain surface before recordings. Thirty minutes after probe insertions, data acquisition started.

Data are acquired at 30 kHz, Intan headstages, and the OpenEphys acquisition system. Each trial recording was triggered using a TTL signal. Raw data were 300 Hz low-pass filtered and downsampled to 1000 Hz for LFP analysis, and were band-pass filtered between 300-6000 Hz for spike clustering using Kilosort (described later the data analysis section). Clusters were manually inspected in Phy to remove noisy units. LFP and clustered spike data were then analyzed using custom scripts in Python.

#### Pupillometry

For some mice, after extracellular recordings, pupil response to vertically drifting gratings (0.04 cycles per degree, 100% contrast, temporal frequencies specified in the main text, including 1.5 Hz, 3 Hz, and 6 Hz) were recorded. The detailed procedure for recording eye movements and pupil size changes are described previously.^47^ Briefly, videos were acquired under infrared illumination using a Thorlabs DCx camera placed at about 28.5 cm away from the mouse eye, using a resolution at 1280x1024. Videos were analyzed post-hoc using a Python computer vision library, OpenCV. Initially, a histogram equalization was utilized to enhance the contrast of the recorded video frames. This helped to ensure that whiskers and small contrast variations did not affect the tracking.

#### Immunohistochemistry

Immunohistochemistry was performed as per our previous protocol.^73^ Briefly, age and sex-balanced WT and Aux-KO mice (n=4-6/group) were anesthetized using isoflurane inhalation and perfused intracardially with 0.9% saline followed by chilled 4% paraformaldehyde prepared in 0.1M phosphate buffer (PB). After post-fixation in the same buffer and cryoprotection in increasing grades of buffered sucrose at 4°C, brains were sectioned (30 μm thick, serial) coronally using a cryostat (Leica CM1850, Germany), collected on gelatinized slides, and stored at -20°C until further use. For immunofluorescence staining, sections were first incubated in 0.5% triton-X 100 (15 mins), followed by incubation in 0.3M Glycine (20 mins). Blocking was performed using 3% goat serum, followed by overnight incubation (4°C) in primary antibodies. Sections were then incubated in Alexa-conjugated secondaries for 3-4 hours, followed by coverslip mounting using an antifade mounting medium with DAPI (H-1000, Vectashield). Coverslips were sealed using nail polish. 1X PBS with 0.1% triton-X 100 was used as both washing and dilution buffer. Below is the list of antibodies used and their dilutions.

**Table:** 

Antibody	Dilution	Manufacturer	RRID
Rabbit Anti-Iba1	1:300	Wako Chemicals (019-19741)	AB_839504
Guinea Pig Anti-GFAP	1:400	Synaptic Systems (173004)	AB_10641162
Guinea Pig Anti-NeuN	1:500	Millipore (ABN90)	AB_11205592
Rabbit Anti-pSer129-α-Syn	1:800	Abcam (ab51253)	AB_869973

#### Microscopy and image analysis

Fluorescent images were acquired using a laser scanning confocal microscope (LSM 800, Zeiss) with a 20X objective (Iba1, GFAP, and pSer129-α-Syn) or using a fluorescence slide scanner (VS200, Olympus) with a 40X objective (NeuN). Appropriate Z-depth was used. FIJI software (National Institute of Health) was used for image analysis as described in Vidyadhara et al.^73^ Briefly, after sum intensity projection, Iba1 and NeuN immunostained images were thresholded using the ‘Otsu’ algorithm and counted using the ‘analyze particles’ function. GFAP+ve astroglial cells were counted manually using the ‘cell counter’ function. The expression intensity for pSer129-α-Synuclein was measured on an 8-bit image as the mean gray value on a scale of 0–255, where ‘0’ refers to minimum fluorescence and ‘255’ refers to maximum fluorescence.

#### Stochastic model for short-term dynamics and multi-vesicular release

In order to better understand and analyze the differences in observed short-term depression, the TM model was utilized.^59^ This model allows for the investigation of the major parameters that contribute to short-term depression, such as U_SE_ and τ_recovery_, which were fitted to the recorded ESPCs for each recorded cell by minimizing mean square errors. Additionally, the RRP properties were estimated with the SMN approach.^41^^,^^42^ This approach allowed the approximation of RRP size and replenishment rate, which were quantified by the intercept and slope of fitted lines on the steady-state cumulative ESPC peak amplitudes.^41^ The example scripts for these modeling analyses were available on a digital repository.

#### Network model of the V1

The V1 cortex model was built by simulating 3200 excitatory neurons and 800 inhibitory neurons using a leaky integrate-and-fire model and a simplified Tsodyks-Markram synapse model.^100^^,^^101^^,^^102^ The membrane potentials (v) of V1 neurons were expressed by the following equations. The values and descriptions of modeling parameters are listed in table below.

$$C_{m}\frac{dv}{dt}=g_{L}\left(E_{l}-v\right)+g_{e}\left(E_{e}-v\right)+g_{i}\left(E_{i}-v\right)$$

$$\frac{dg_{e,i}}{dt}=-\frac{g_{e,i}}{\tau_{e,i}}$$

To simulate short-term synaptic plasticity, the synaptic transmission was modeled by the dynamics of the neurotransmitter usage per pre-synaptic spike (u) and the fraction of available neurotransmitters (x) described by the following expressions.

$$\frac{du}{dt}=-\frac{u}{\tau_{facil}}$$

$$\frac{dx}{dt}=\frac{1-x}{\tau_{rec}}$$

The synaptic conductances($g_{e,i}$) were dynamically derived at the arrival of pre-synaptic spikes according to the following expressions.

u←u+U(1−u)

r←ux

x←x−r

$$g_{e,i}\leftarrow g_{e,i}+{rw}_{e,i}$$

Neurons in the V1 cortex model received simulated afferent inputs from 3000 thalamocortical neurons that elicited Poisson spike trains with rates ranging from 10 to 20 Hz shaped by a 200-ms Hanning window. The simulation was repeated 10 times using different random seeds. The firing rates were computed by taking the average firing rates of all simulated V1 neurons and normalized by substracting the mean and dividing by the standard deviations of the baseline firing rates and then filtered by a Gaussian filter with a 10-ms kernel. The deficits in synaptic plasticity were modeled by adjusting $\tau_{facil}$ (STP) and $\tau_{rec}$ (STD) (Figure S3).

**Table:** 

Parameter	value	Parameter	value
$C_{m}$, membrane capacitance	350 pF	$\tau_{e}$, excitatory time constant	5 ms
$E_{l}$, leak reversal potential	-72 mV	$\tau_{i}$, inhibitory time constant	10 ms
$E_{e}$ , excitatory reversal potential	0 mV	$\tau_{r}$, refractory period	1 ms
$E_{i}$ , inhibitory reversal potential	-80 mV	$w_{e}$, excitatory synaptic weight	1 nS
$g_{l}$ , leaky conductance	20 nS	$w_{i}$, inhibitory synaptic weight	5 nS
$V_{th}$ , threshold voltage)	-55 mV	U, rest synaptic release probability	0.2
$p_{in}$, input connectivity	0.04	$\tau_{facil}$, facilitation time constant	20 ms (WT) 10 ms (AuxKO)
$p_{e}$, excitatory neuron connectivity	0.025	$\tau_{rec}$, recovery time constant	300 ms (WT) 200 ms (AuxKO)
$p_{i}$, inhibitory neuron connectivity	0.05	dt, sampling time step	0.1 ms

### Quantification and statistical analysis

#### *Ex vivo* data analysis

Evoked EPSC values were the average of all the trails for recording from each neuron. For the data from the paired-pulse stimulation protocol, the paired-pulse ratio (PPR) was calculated: second evoked EPSC/first evoked EPSC. For multiple pulse stimulation protocols, the ratio of each evoked EPSC to the first evoked EPSC was calculated. Different statistic tests were applied subject to the distributions of parameters. The Student’s student’s t-test was used if the data from two groups are normally distributed verified by the Shapiro-Wilk test, and the variance is comparable (variance ratio less than 2). If two groups of data are normally distributed while the variance is not relatively equal, Welch’s t-test was done instead. Mann-Whitney U test was used when data is not normally distributed, and variance is unequal.

#### *In vivo* data analysis

*In vivo* data analysis is performed using customized scripts in Python, similarly as previously described.^28^^,^^45^^,^^47^^,^^103^ For LFP analysis, the LFP with the largest amplitude within 50-150 ms post- visual stimulation onset between 300 to 500 μm below the brain surface was identified as the layer 4 LFP for each columns of channels of each probe (3 layer 4 LFPs per recording). Then, all layer 4 LFPs were used for further analyses. For the LFP analysis in the oddball paradigm, each layer 4 LFP were trial averaged first, then the amplitudes of the LFP within the time window of interests were compared between groups. For the visual chirp experiment, the spectrogram (time frequency analysis) of each layer 4 LFP was computed from 0.5-80 Hz by convolving with morlet wavelets with varying cycles from two to twenty-two (MNE). The LFP power was baseline normalized by being divided by the averaged power within 400 ms before the stimulus onset. The baseline normalized power was then averaged across trials and was converted to decibels (dB) for each LFP. The power in decibels within time windows of interests was extracted and averaged for quantifications.

For single unit analyses, 300-6000 Hz bandpass filtered data were used for spike clustering using Kilosort algorithm 1 or 2,^104^ with spike detection threshold at 4 standard deviations. Following Kilosort spike clustering, units were inspected in Phy GUI,^105^ similarly as previously described.^28^^,^^45^^,^^47^^,^^103^ Units were labeled as good units if they 1) had median inter-spike intervals larger than 1 ms (absolute refractory period); 2) had typical spike waveforms showing peak at the center but not at the beginning or the end (noise-like waveforms); 3) had spike detected at channels that were geometrically close to each other. After the manual inspection, good units from all layers with larger than 0.5 Hz mean firing rates were included in the analysis, and the number of units was stated in the figure legends. For each unit, the spike histogram was smoothed using a gaussian kernel (50 ms as one standard deviation) to get trial averaged firing rates (FR) over time. For baseline normalized stimulus locked response, it was calculated as (FR-FR_0_)/std(FR_0_), where FR_0_ is the averaged firing rate within 0.2 s before the stimulus onset. The quantifications were done on either firing rates or baseline normalized responses averaged within time windows of interests. For gaussian function fitting in the orientation selectivity analysis, six response values were fitted into a gaussian function ($A*e^{\left(\frac{{-\left(x-\mu \right)}^{2}}{2\sigma^{2}}\right)}+b$) using Scipy curve_fit function. The amplitude (A), center (μ), standard deviation (σ), offset (b) were extracted for parameterize the tuning curve. Good fits were defined as the fits with R^2^ larger than 0.95. For unit analysis of the post-experience oscillations, firing rate z-scores were first computed. Then, the post-experience oscillation peaks were identified as local extreme z-scores if they were at least 100 ms apart with at least 0.5 in amplitude. To quantify the oscillation frequencies of the units, units that had at least two oscillation peaks were first selected, and the adjacent peak durations were inversed and averaged for each unit to get the oscillation frequency of the unit.

Statistical tests were performed using statistical packages including SciPy, and Pingouin. Data normality was tested using the Shapiro-Wilk test first. For normally distributed data, t-tests were used. For non-normally distributed data, Mann–Whitney U tests or Kolmogorov–Smirnov 2 sample tests were used. P values of multiple comparisons were corrected using the FDR-BH method.

#### Pupil video analysis

All videos were analyzed with DeepLabCut, an open-sourced video processing tool.^50^ Here, we trained a convolutional neural network (ResNet50) to detect pupil coordinates. A training set of frames from various mice under various lighting conditions was used where we manually selected four coordinates outlining the pupil (North, South, East, and West). A circle was fitted to these coordinates using a least-square optimization in Python. Additionally, a center point for the pupil was found based on this fitted circle. Outlier data points were excluded by thresholding pupil diameter to be in the range of 10-50 pixels. After the pupil dynamics had been recorded, the output raw trace data was initially median filtered with a window size of 11. Saccades were detected using PyGaze, an open-source toolbox for eye tracking. Under this function, saccades are defined as consecutive samples with an inter-sample velocity greater than both a velocity (40 pixels/second) and an acceleration threshold (340 pixels/second/second). Both the start and end time points of a saccade are found using these two thresholds where the start and end of the saccade are defined as having velocity and acceleration values greater than and less than the thresholds, respectively. Furthermore, trials with less than two detected saccades were excluded. Quantification analysis of the saccades included looking at the total number of saccades (per session and during periods of stimulus presentation and a control grey screen), amplitude (x-dimension distance traveled), and maximum velocity within the start-end time window for each saccade.

## Data Availability

• The data and code presented in this paper will be made available by the lead contact upon request. • Any additional information is available from the lead contact upon request. • This paper does not report original code.
