# Supplementary material for: Impaired pre-synaptic plasticity and visual responses in auxilin-knockout mice
Source: iScience. 2023 Sep 6;26(10):107842. doi: 10.1016/j.isci.2023.107842 (PMC10520332; doi:10.1016/j.isci.2023.107842)
Supplement: Document S1. Figures S1–S16 [file mmc1.pdf]

iScience, Volume *26*

Supplemental information

Impaired pre-synaptic plasticity and visual responses in auxilin-knockout mice

Xi Cheng, Yu Tang, D.J. Vidyadhara, Ben-Zheng Li, Michael Zimmerman, Alexandr Pak, Sanghamitra Nareddula, Paige Alyssa Edens, Sreeganga S. Chandra, and Alexander A. Chubykin

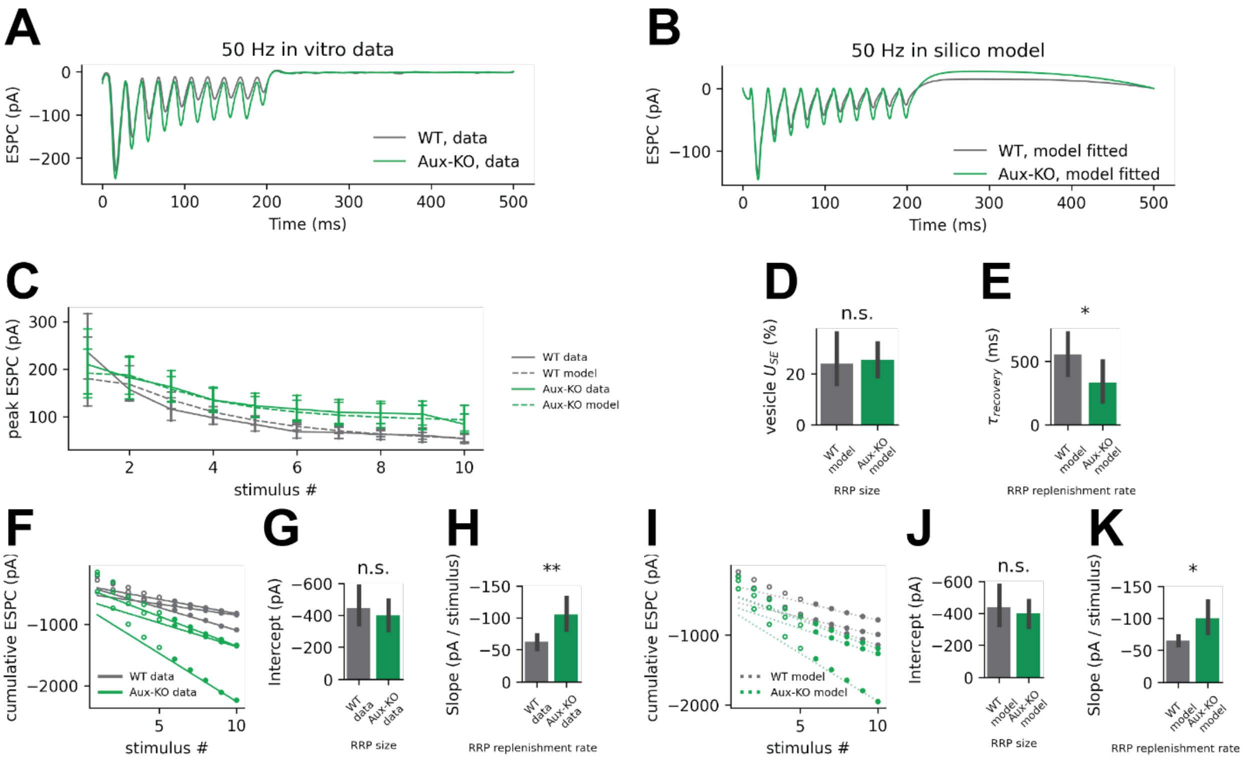

Figure S1. 50 Hz Multiple-pulse responses in Aux-KO mice following repetitive stimulation, related to Figure 3.

(A) Mean trace of ex vivo ESPC data in the WT group and Aux-KO group.
(B) Mean in-silico model simulation of ESPCs under 20 Hz 10 pulses in the WT group and Aux-KO group. Raw traces were processed as described in (A).
(C) Peak absolute ESPC amplitude at each stimulus pulse. The error bar indicates SEM.
(D) Fitted U_{SE} from the TM model.
(E) Fitted $\tau_{recovery}$ from the TM model.
(F) Estimation of RRP properties in ex vivo ESPC data with the SMN approach. Steady-state cumulative ESPC peak amplitudes (filled circles) were linearly fitted.
(G) Intercept and RRP size from SMN approach.
(H) Slope and RRP replenishment rates from SMN approach.
(I-K) Replication of SMN approach (F-H) in in-silico results in fitted TM-model.

Data were presented as mean ± SEM. *p<0.05, **p<0.01, n.s. - not significant.

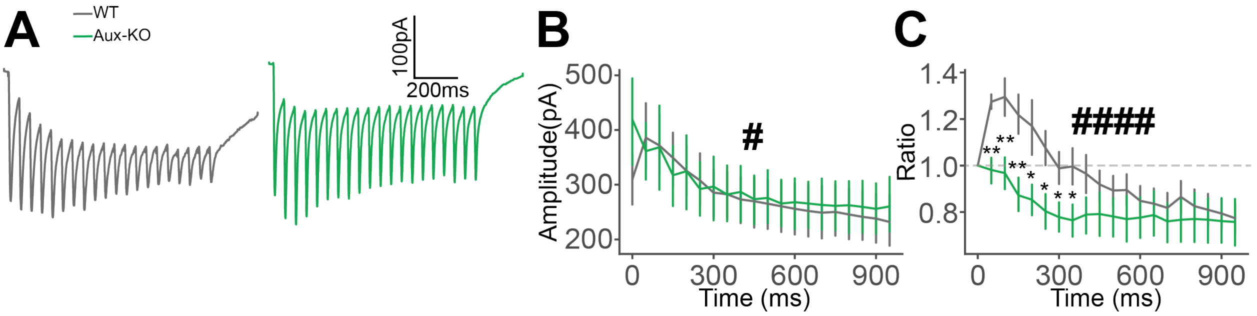

Figure S2. Multiple-pulse responses in Aux-KO mice following repetitive stimulation, related to Figure 2.

(A) Average of multiple EPSC traces from prolonged high-frequency stimulation of WT and Aux-KO.

(B) Average amplitudes upon each stimulus of multiple-pulse training (WT: N=14 cells, 6 mice, Aux-KO: N=13 cells, 6 mice). Two-way ANOVA with Tukey's post hoc.
(C) Average MPRs upon each pulse (WT: N=14 cells, 6 mice, Aux-KO: N=13 cells, 6 mice). Two-way ANOVA with Tukey's post hoc.

Data were presented as mean ± SEM. Two-way ANOVA: #p<0.05, ####p<0.0001. Tukey's post hoc: *p<0.05, **p<0.01

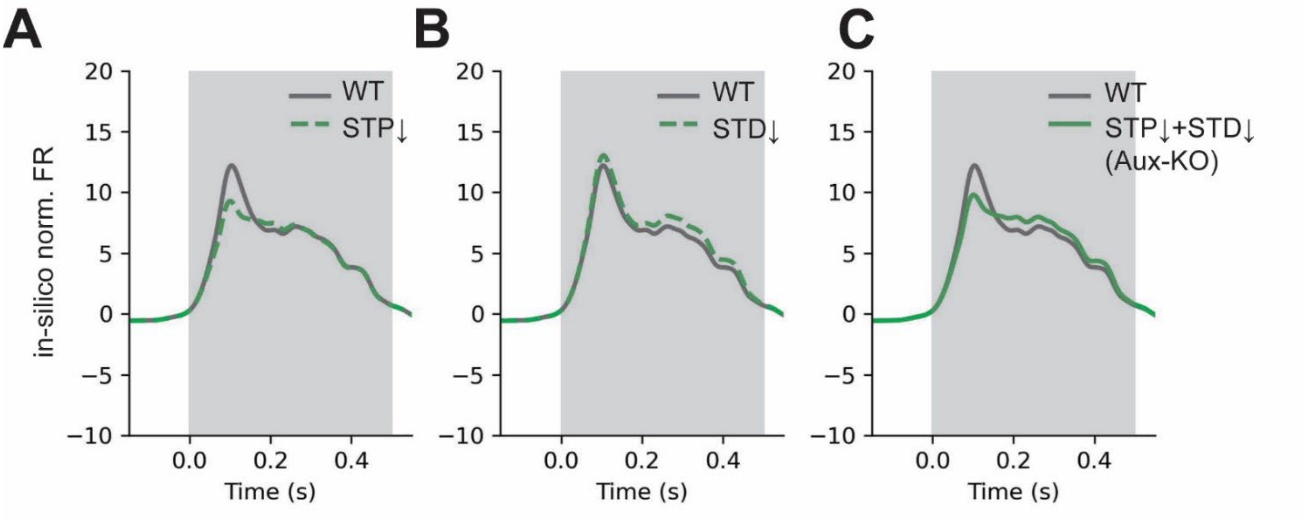

Figure S3. *In silico* simulation of mouse V1 activity in WT and Aux-KO mice, related to Table 1.

(A) *In silico* simulated normalized firing rates (norm. FR) of WT unit populations and unit populations with reduced STP.
(B) *In silico* simulated normalized firing rates (norm. FR) of WT unit populations and unit populations with reduced STD.
(C) *In silico* simulated normalized firing rates (norm. FR) of WT unit populations and unit populations with reduced STP and reduced STD (Aux-KO).

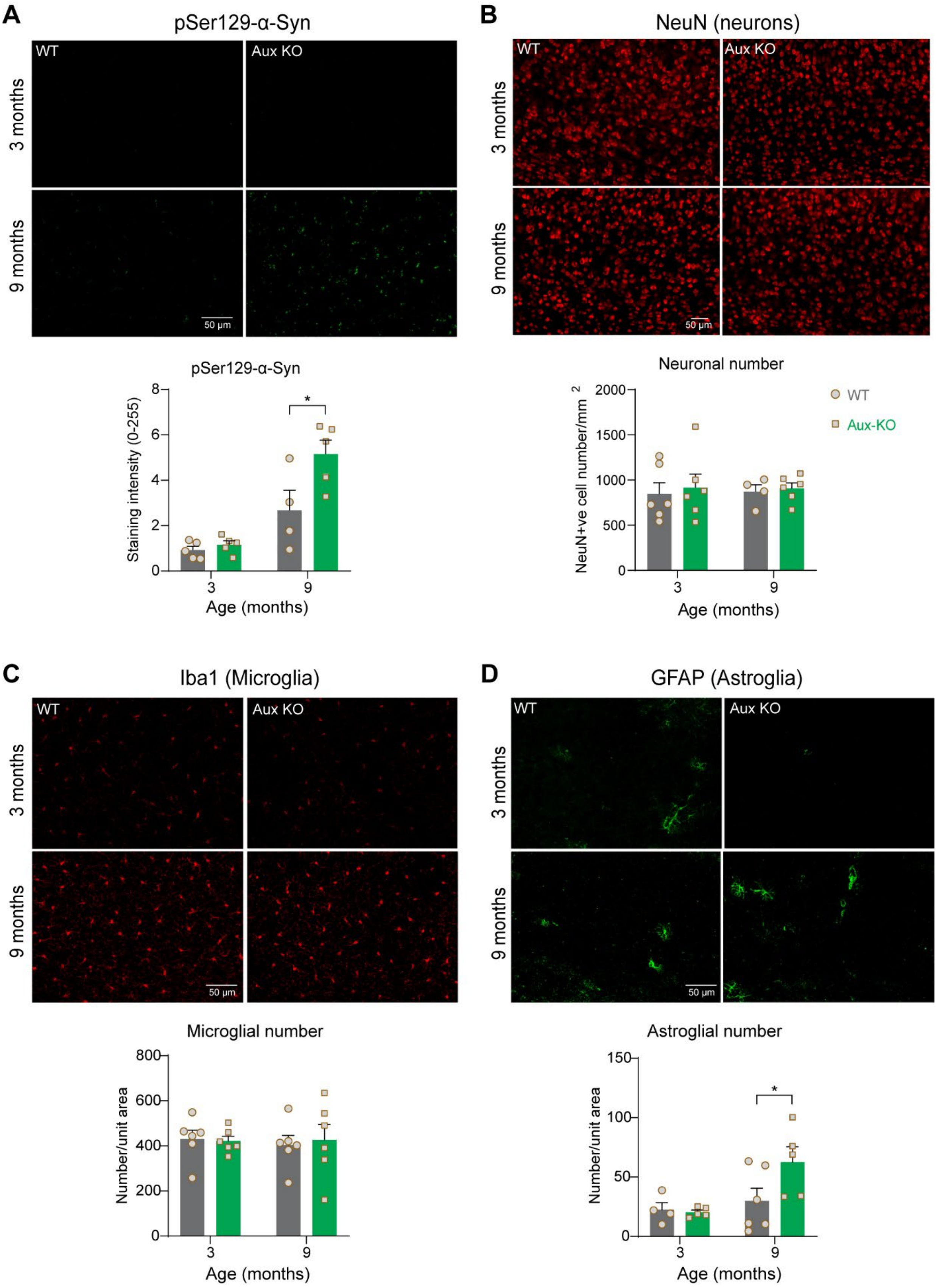

Figure S4. Aux-KO mice display α-synuclein pathology at an older age, related to Figure 1.

(A) Representative images and graph showing increased pSer129-α-synuclein expression in the visual cortex at 9 months (WT: N=5 mice, Aux-KO: N=5 mice), but not at 3 months old Aux-KO mice (WT: N=5 mice, Aux-KO: N=5 mice).

(B) Representative images of NeuN (red), and the quantitation graph show no changes in the numbers of neurons in the visual cortex of Aux-KO mice (3-month-old: WT: N=6 mice, Aux-KO: N=6 mice; 9-month-old: WT: N=4 mice, Aux-KO: N=6 mice).

(C) Representative images of Iba1 +ve microglia (red), and the quantitation graph showing no microgliosis in the visual cortex of Aux-KO mice (3-month-old: WT: N=6 mice, Aux-KO: N=6 mice; 9-month-old: WT: N=6 mice, Aux-KO: N=6 mice).

(D) Representative images of GFAP +ve astroglia (green), and the quantitation graph showing increased astrogliosis in the visual cortex of Aux-KO mice at 9 months (3-month-old: WT: N=4 mice, Aux-KO: N=5 mice; 9-month-old: WT: N=6 mice, Aux-KO: N=5 mice).

Scale: 50 μm

Statistics: Student's t-test with Welch's correction, *$p<0.05$, data expressed as mean +/- SEM.

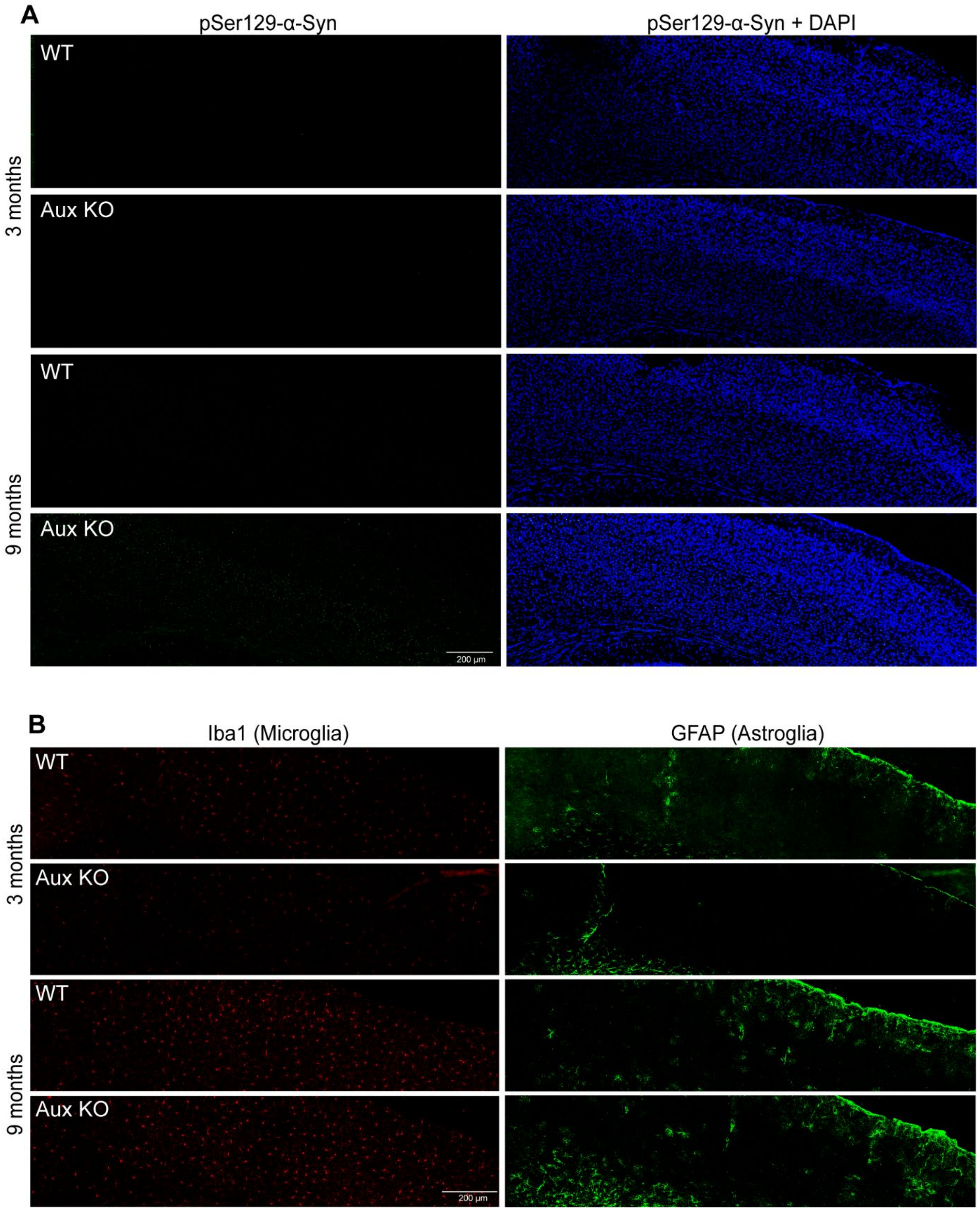

Figure S5. Representative low magnification images of visual cortex immunostained for (A) pSer129-α-Syn (pathological α-synuclein) and (B) Iba1 (microglia) and GFAP (astroglia). Scale: 200 µm, related to Figure 1.

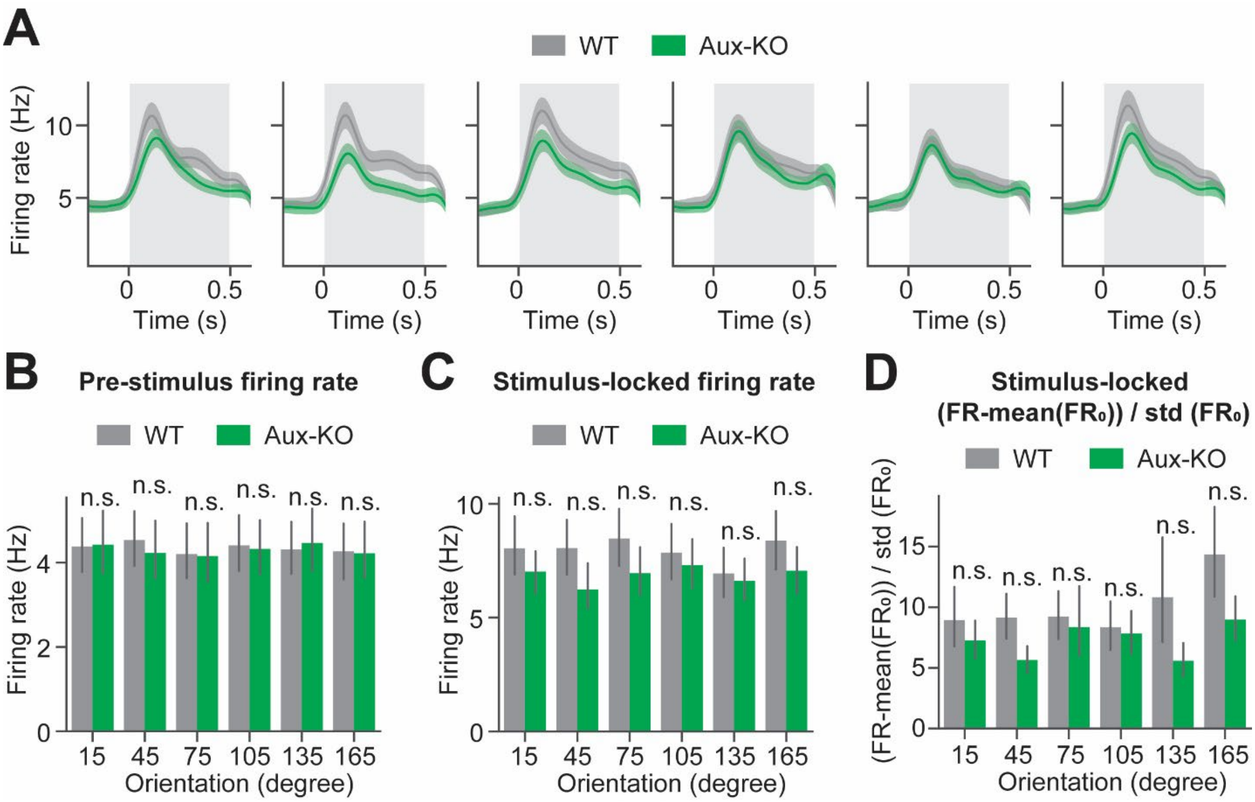

Figure S6. Spontaneous firing rates, stimulus-locked firing rates, and baseline normalized stimulus-locked responses of unit populations were comparable between WT and Aux-KO mice, related to Figure 4.

(A) Unit population firing rates over time. The gray shade represents the visual stimulation time window. WT: N=342 units, 7 mice; Aux-KO: N=367 units, 10 mice. Data were presented as mean ± SEM.

(B) Pre-stimulus (-0.2 s to 0 s) firing rates plotted in a bar plot. The error bar represents mean ± 95% confidence interval (CI). WT: N=342 units, 7 mice; Aux-KO: N=367 units, 10 mice. Units' firing rates were tested using Mann-Whitney U tests with FDR-BH correction.

(C) Stimulus-locked (0 – 0.5 s) firing rates plotted in a bar plot. The error bar represents mean ± 95% CI. WT: N=342 units, 7 mice; Aux-KO: N=367 units, 10 mice. Units' firing rates were tested using Mann-Whitney U tests with FDR-BH correction.

(D) Baseline normalized stimulus-locked responses are plotted in a bar plot. The error bar represents the mean ± 95% CI. WT: N=342 units, 7 mice; Aux-KO: N=367 units, 10 mice. Units' firing rates were tested using Mann-Whitney U tests with FDR-BH correction.

n.s. - not significant.

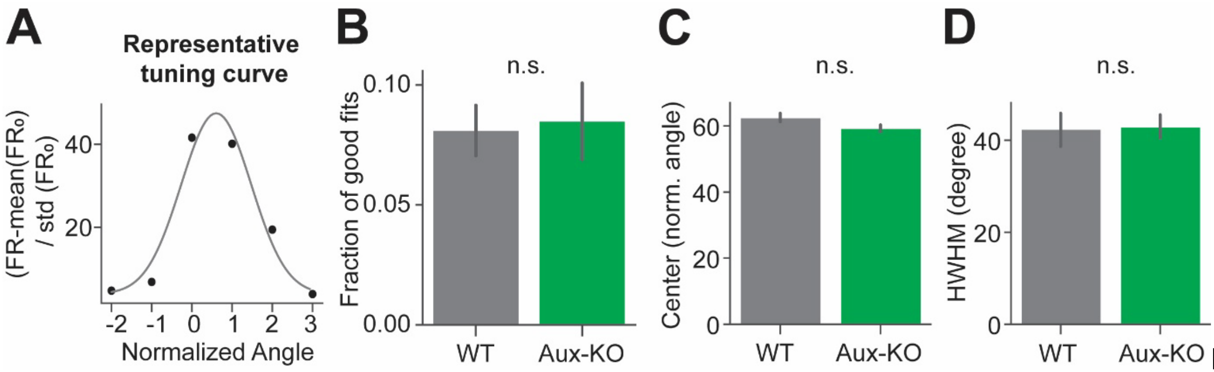

Figure S7. Parameterization of tuning curves using Gaussian fits, related to Figure 4.

(A) A representative Gaussian fit.
(B) Fractions of good Gaussian fit (R^2>0.95) are plotted in a bar plot. WT: N=7 mice; Aux-KO: N=10 mice. The error bar represents mean ± SEM. Fractions of good fits were compared using t-test with Welch's correction.
(C) Centers of good Gaussian fit (R^2>0.95) plotted in a bar plot. WT: N=8 units; Aux-KO: N=8 units. The error bar represents mean ± SEM. Centers of good fits were compared using t-test with Welch's correction.
(D) HWHMs of good Gaussian fit (R^2>0.95) plotted in a bar plot. WT: N=8 units; Aux-KO: N=8 units. The error bar represents mean ± SEM. Centers of good fits were compared using t-test with Welch's correction.

n.s. - not significant.

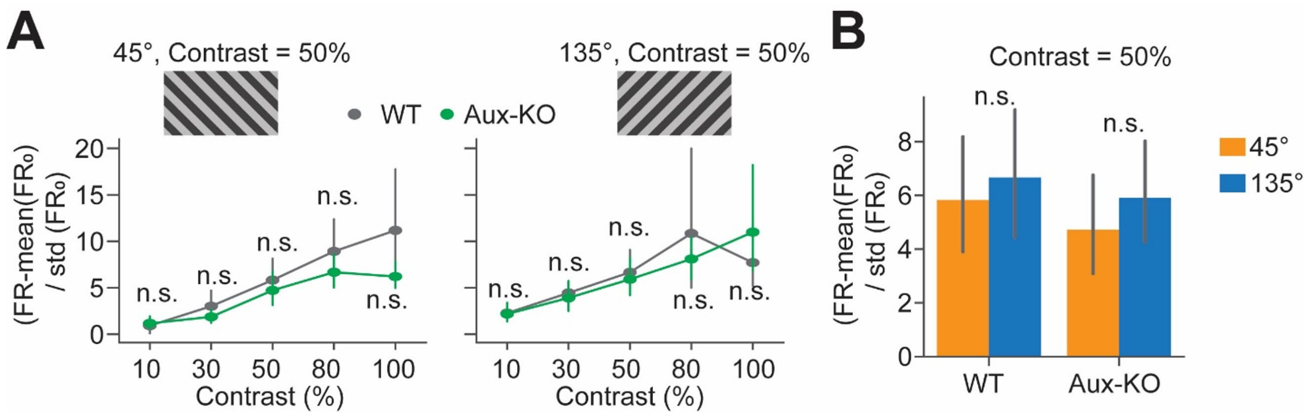

Figure S8. Contrast sensitivity is similar in Aux-KO and WT, related to Figure 4.

(A) Stimulus-locked responses (baseline normalized) to 45° (left) and 135° (right) at five contrasts. 45°: WT: N=71 units, 3 mice; Aux-KO: N=203 units, 5 mice. Units' stimulus-locked responses were compared between WT and Aux-KO at each contrast using Mann-Whitney U tests with FDR-BH correction. Data were presented as mean ± 95% CI.
(B) Stimulus-locked responses to 45° (left) and 135° (right) at 50% contrast plotted in a bar plot. Units' stimulus-locked responses were compared between WT and Aux-KO at each contrast using Mann-Whitney U tests with FDR-BH correction. Data were presented as mean ± 95% CI.

n.s. - not significant.

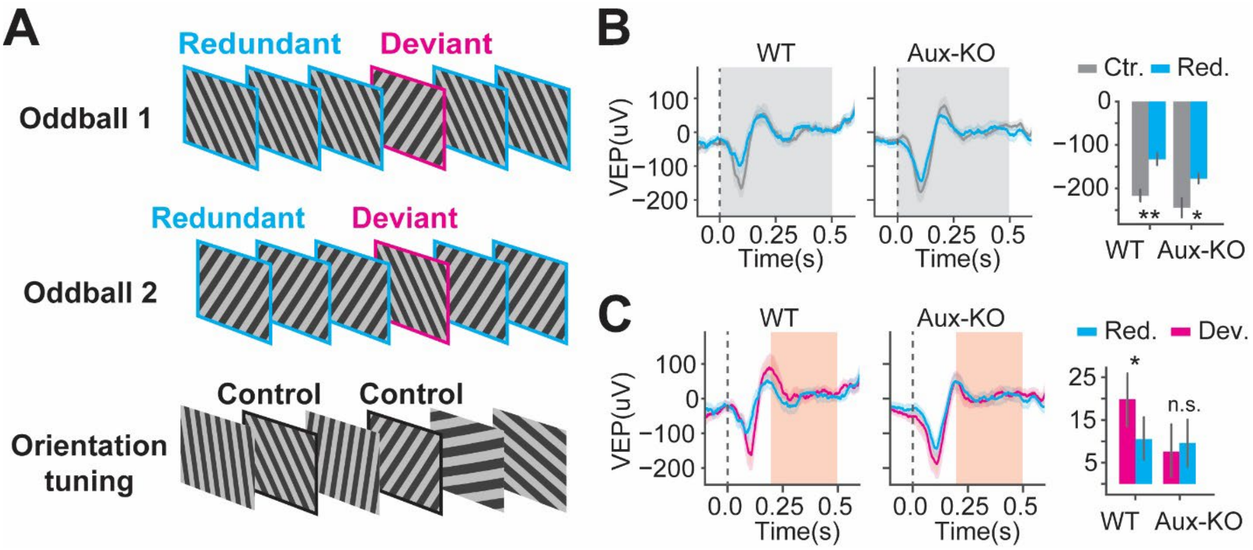

Figure S9. Visual mismatch response was reduced in Aux-KO mice, related to Figure 6.

(A) The oddball paradigm. Two orientations were presented in a visual stimulation sequence. The redundant orientation was presented in 90% of the trials, and the deviant orientation was presented in 10% of the trials. The redundant and deviant orientations were counterbalanced in two visual stimulation sequences. The same two orientations were presented in another sequence of orientations with equal appearance frequency as controls.

(B) Visually evoked potential in V1 layer 4 in responses to the control and redundant stimuli. The largest negativity within the visual stimulation time window of each recording was quantified. WT: N=18 LFPs, 6 mice; Aux-KO: N=30 LFPs, 10 mice. LFPs were compared using Mann-Whitney U tests with FDR-BH correction.

(C) Visually evoked potential in V1 layer 4 in responses to the deviant and redundant stimuli. The mean potential within 0.2-0.5 s after the visual stimulation onset of each recording was quantified. WT: N=18 LFPs, 6 mice; Aux-KO: N=30 LFPs, 10 mice. LFPs were compared using Mann-Whitney U tests with FDR-BH correction.

Data were presented as mean ± SEM. *p<0.05, **p<0.01, n.s. not significant.

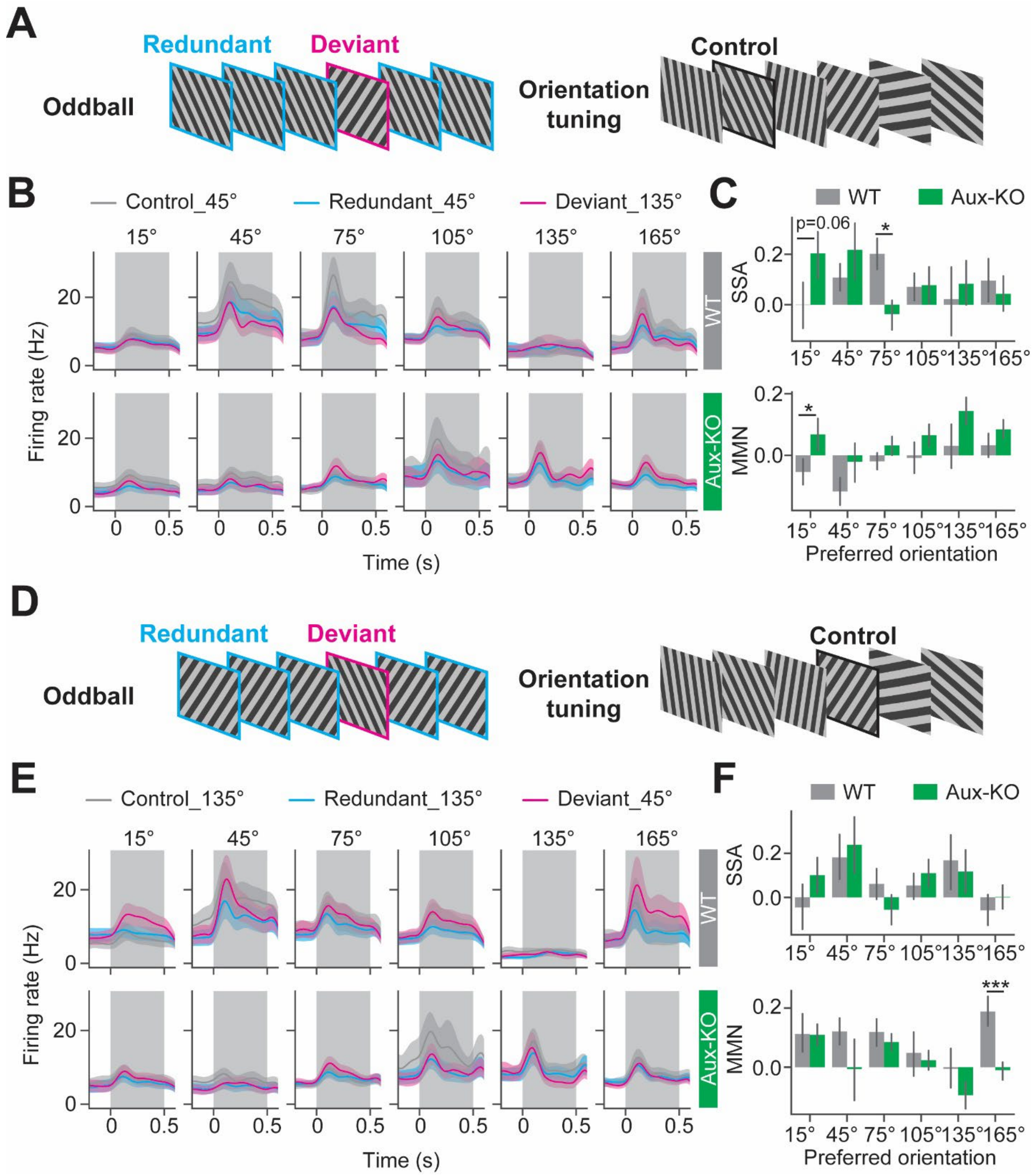

Figure S10. V1 unit activity in the visual mismatch paradigm in WT and Aux-KO mice, related to Figure 6.

(A) The oddball sequence 1 with the 45° orientation as the redundant stimulus and the 135° orientation as the deviant stimulus. The 45° orientation in the control sequence was used as the control.

(B) Firing rates of units that have smaller than 0.2 orientation selectivity index (OSI) in response to the redundant, deviant, and control stimuli in the oddball sequence 1. Units were separated into six groups based on their preferred orientations indicated on top. WT: 15°: N=20 units, 45°: N=16 units, 75°: N=23 units, 105°: N=30 units, 105°: N=8 units, 105°: N=25 units; Aux-KO: 15°: N=19 units, 45°: N=15 units, 75°: N=37 units, 105°: N=28 units, 105°: N=19 units, 105°: N=31 units. Data were presented as mean ± SEM.

(C) Stimulus-specific adaptation (SSA, top) and mismatch negativity (MMN, bottom) are plotted in bar plots. Stimulus-specific adaptation: the mean firing rate within the control stimulus time window subtracted by the mean firing rate within the redundant stimulus time window. Mismatch negativity: the mean firing rate within the deviant stimulus time window subtracted by the mean firing rate within the redundant stimulus time window. WT: 15°: N=20 units, 45°: N=16 units, 75°: N=23 units, 105°: N=30 units, 105°: N=8 units, 105°: N=25 units; Aux-KO: 15°: N=19 units, 45°: N=15 units, 75°: N=37 units, 105°: N=28 units, 105°: N=19 units, 105°: N=31 units. Data were presented as mean ± SEM. SSAs (or MMNs) of units within each orientation group were compared using Mann-Whitney U tests.

(D) The oddball sequence 2 with the 135° orientation as the redundant stimulus and the 45° orientation as the deviant stimulus. The 135° orientation in the control sequence was used as the control.

(E) Firing rates of units that have smaller than 0.2 orientation selectivity index (OSI) in response to the redundant, deviant, and control stimuli in the oddball sequence 2. Units were separated into six groups based on their preferred orientations indicated on top. WT: 15°: N=17 units, 45°: N=13 units, 75°: N=15 units, 105°: N=23 units, 105°: N=6 units, 105°: N=20 units; Aux-KO: 15°: N=18 units, 45°: N=15 units, 75°: N=37 units, 105°: N=27 units, 105°: N=19 units, 105°: N=30 units. Data were presented as mean ± SEM.

(F) Stimulus-specific adaptation (SSA, top) and mismatch negativity (MMN, bottom) are plotted in bar plots. WT: 15°: N=17 units, 45°: N=13 units, 75°: N=15 units, 105°: N=23 units, 105°: N=6 units, 105°: N=20 units; Aux-KO: 15°: N=18 units, 45°: N=15 units, 75°: N=37 units, 105°: N=27 units, 105°: N=19 units, 105°: N=30 units. Data were presented as mean ± SEM. SSAs (or MMNs) of units within each orientation group were compared using Mann-Whitney U tests.

*p<0.05, **p<0.01, ***p<0.01, n.s. - not significant. See the extended table for detailed statistics.

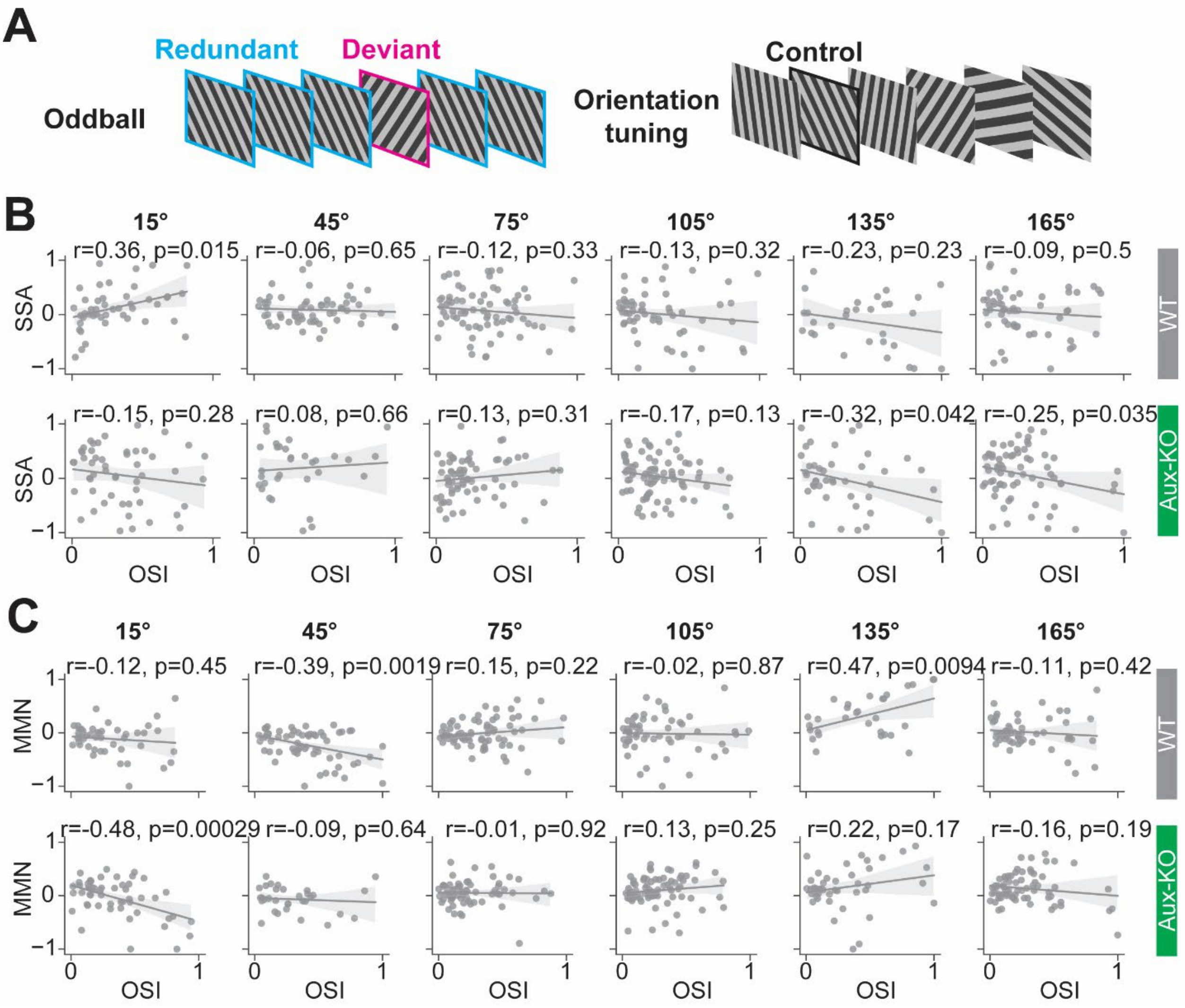

Figure S11. Correlations between OSIs and stimulus-specific adaptation (SSA) and correlations between OSIs and mismatch response (MMN) in the oddball sequence 1, related to Figure 6.

(A) The oddball sequence 1 with the 45° orientation as the redundant stimulus and the 135° orientation as the deviant stimulus. The 45° orientation in the control sequence was used as the control.

(B) Correlations between unit OSIs and SSA responses. All units were separated into six groups based on their preferred orientations indicated on top. Pearson's r and statistical significance of the linear regression were indicated in the graphs.

(C) Correlations between unit OSIs and MMN responses. All units were separated into six groups based on their preferred orientations indicated on top. Pearson's r and statistical significance of the linear regression were indicated in the graphs.

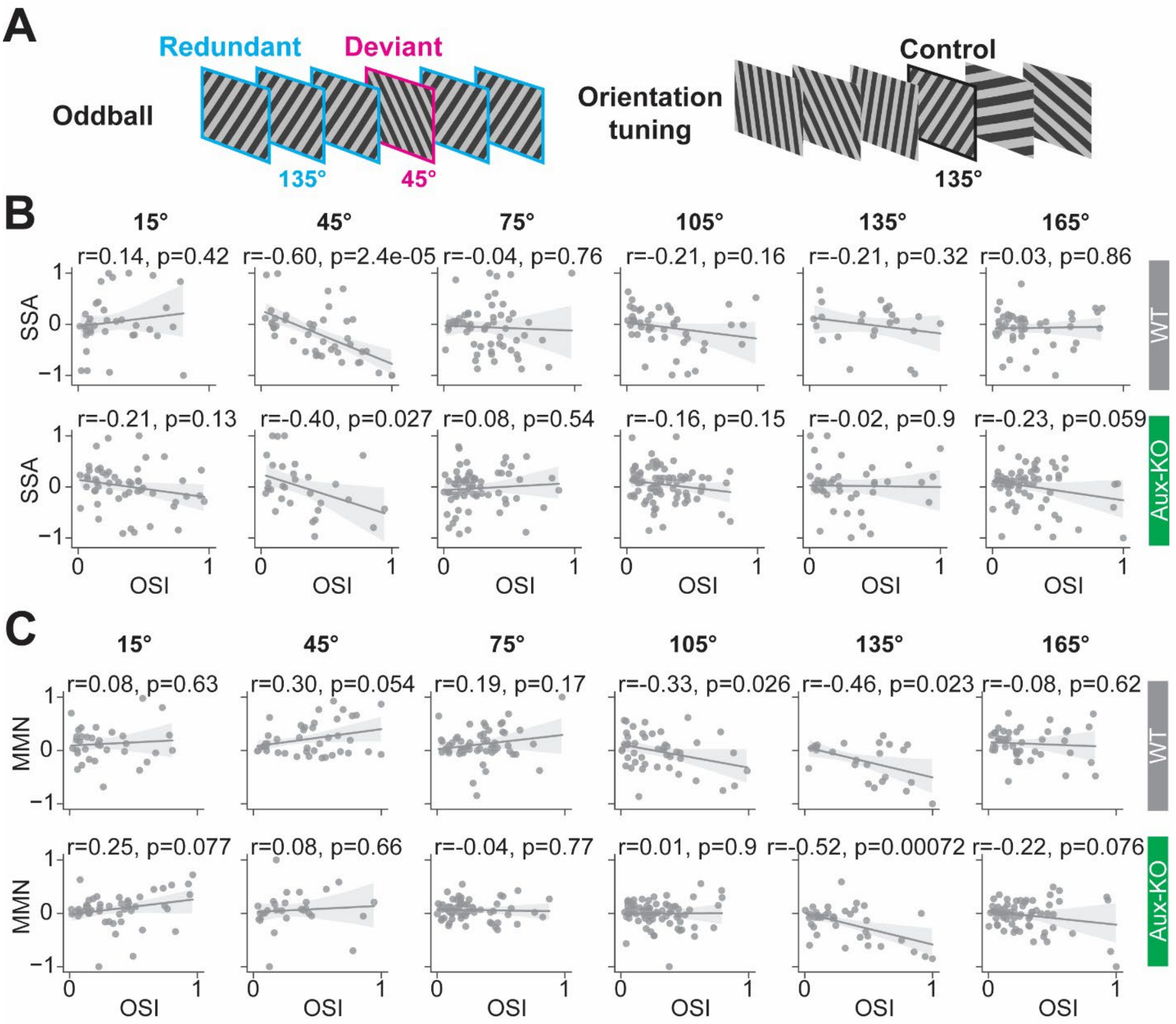

Figure S12. Correlations between OSIs and stimulus-specific adaptation (SSA) and correlations between OSIs and mismatch response (MMN) in the oddball sequence 2, related to Figure 6.

(A) The oddball sequence 2 with the 135° orientation as the redundant stimulus and the 45° orientation as the deviant stimulus. The 135° orientation in the control sequence was used as the control.

(B) Correlations between unit OSIs and SSA responses. All units were separated into six groups based on their preferred orientations indicated on top. Pearson's r and statistical significance of the linear regression were indicated in the graphs.

(C) Correlations between unit OSIs and MMN responses. All units were separated into six groups based on their preferred orientations indicated on top. Pearson's r and statistical significance of the linear regression were indicated in the graphs.

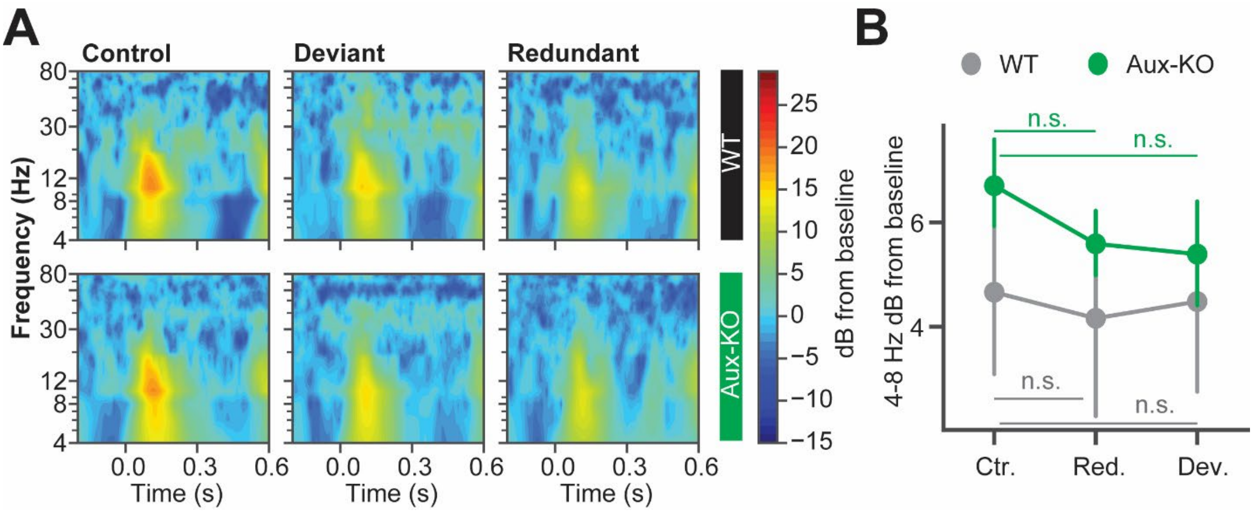

Figure S13. Time-frequency analysis of layer 4 LFPs in WT and Aux-KO mice in the visual mismatch paradigm, related to Figure 6.

(A) Averaged baseline normalized time-frequency spectrograms of layer 4 LFPs in WT and Aux-KO mice.

(B) Mean 4-8 Hz power of layer 4 LFPs within the visual stimulation time window in WT and Aux-KO mice. WT: Control: N=18 LFPs, 6 mice, Deviant: N=21 LFPs, 7 mice, Redundant: N=21 LFPs, 7 mice; Aux-KO: N=30 LFPs, 10 mice, Deviant: N=30 LFPs, 10 mice, Redundant: N=30 LFPs, 10 mice. The error bar represents the mean ± 95% confidence interval (CI). 4-8 Hz power of LFPs was tested using Mann-Whitney U tests with FDR-BH correction.

n.s. - not significant.

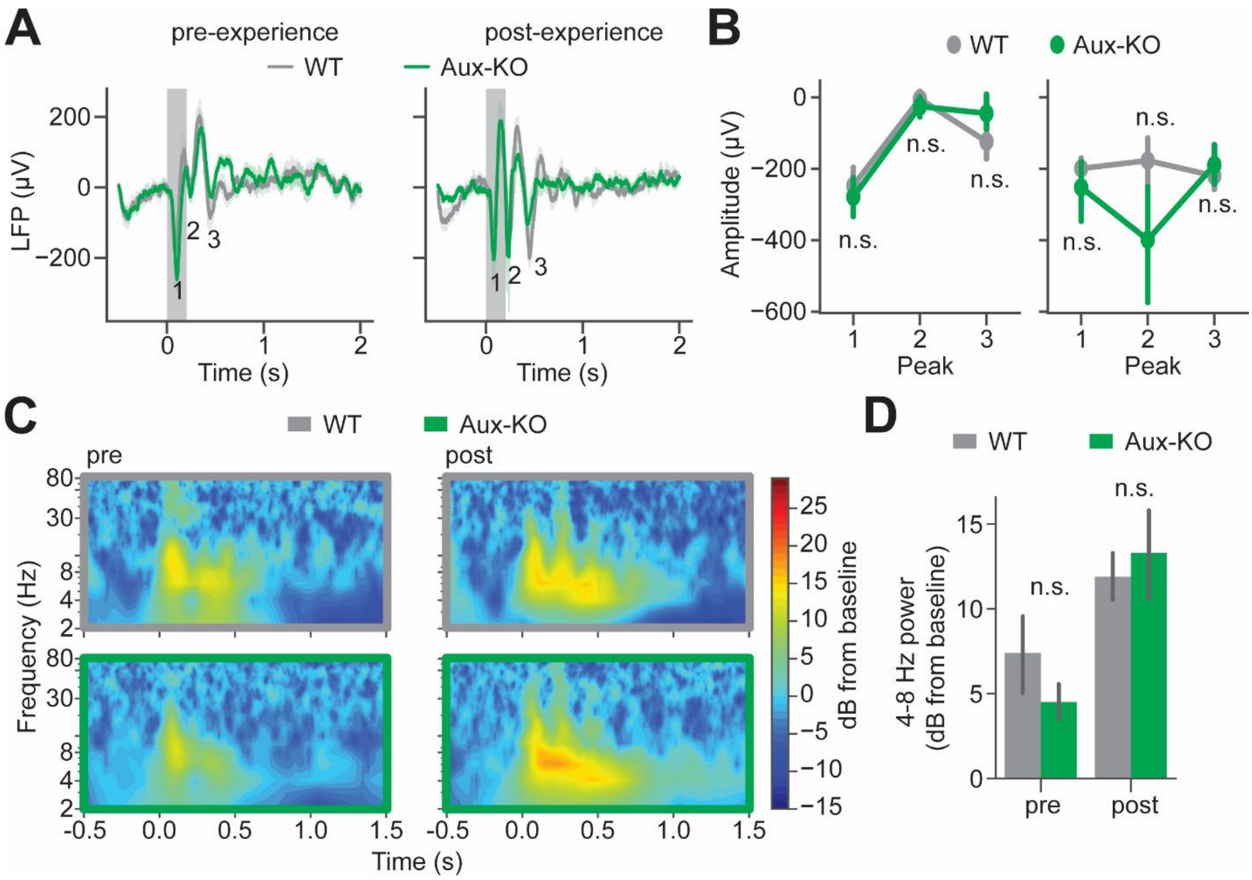

Figure S14. 4-8 Hz power of layer 4 LFPs in Aux-KO and WT before and after the visual experience, related to Figure 7.

(A) Layer 4 local field potentials (LFPs) in WT and Aux-KO in pre- and post-visual experience. The gray shade represents the visual stimulation time window. LFP peaks were indexed. WT: pre: N=21 LFPs, 7 mice, post: N=21 LFPs, 7 mice; Aux-KO: pre: N=30 LFPs, 10 mice, post: N=27 LFPs, 9 mice. Data were presented as mean ± SEM.

(B) Layer 4 LFP peak amplitudes in pre- and post-visual experience. The error bar represents mean ± 95% confidence interval (CI). WT: pre: N=21 LFPs, 7 mice, post: N=21 LFPs, 7 mice; Aux-KO: pre: N=30 LFPs, 10 mice, post: N=27 LFPs, 9 mice. LFPs were tested using Mann-Whitney U tests with FDR-BH correction.

(C) Averaged baseline normalized time-frequency spectrograms of layer 4 LFPs in WT and Aux-KO mice.

(D) 4-8 Hz baseline normalized power plotted in a bar plot. The error bar represents mean ± 95% CI. WT: pre: N=21 LFPs, 7 mice, post: N=21 LFPs, 7 mice; Aux-KO: pre: N=30 LFPs, 10 mice, post: N=27 LFPs, 9 mice. LFP's 4-8 Hz powers were tested using Mann-Whitney U tests with FDR-BH correction.

n.s. - not significant.

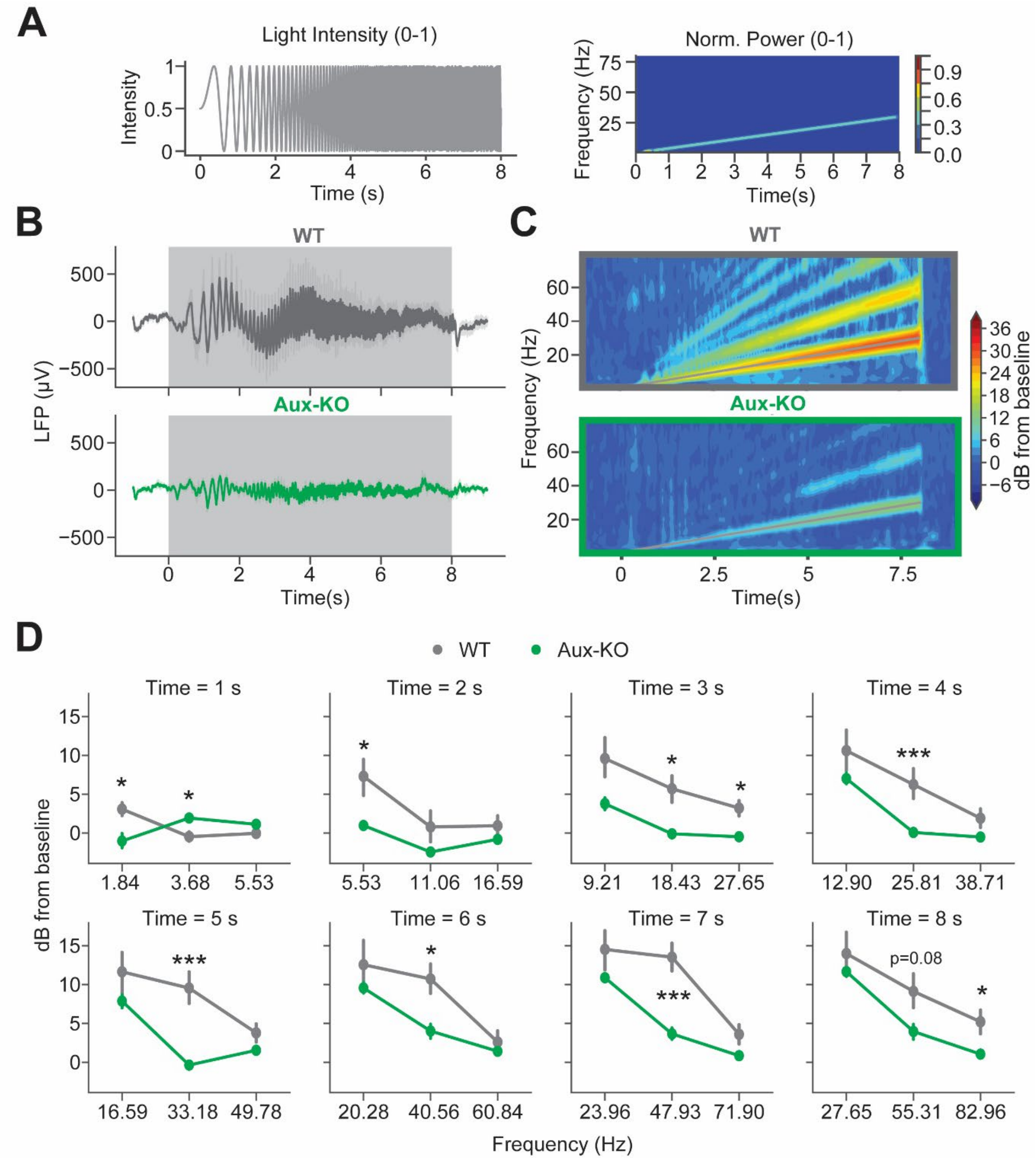

Figure S15. Visually evoked potentials of Aux-KO mice had decreased power in response to a 0.5 – 30 Hz visual flicker chirp at the stimulation frequencies and harmonic frequencies, related to Figure 4.

(A) Left: Visual flicker chirp light intensities over time. The light intensity was normalized from zero to one (0 is black, 0.5 is gray, 1 is white). Right: A spectrogram of the chirp light intensity revealing frequency change from 0.5 to 30 Hz over time.

(B) Averaged layer 4 LFPs in response to the flicker chirp in WT (top) and Aux-KO (bottom). The gray shaded area represents the visual stimulation time window.

(C) Averaged baseline normalized spectrograms of layer 4 LFPs in WT (top) and Aux-KO (bottom). WT: N=12 LFPs, 4 mice; Aux-KO: N=12 LFPs, 4 mice.

(D) Power is measured at the stimulation frequency, the first harmonic frequency, and the second harmonic frequency plotted in point plots at multiple time points. The error bar represents mean ± SEM. LFP values were tested using Mann-Whitney U tests with FDR-BH corrections.

***p<0.001, **p<0.01,*p<0.05, n.s. - not significant.

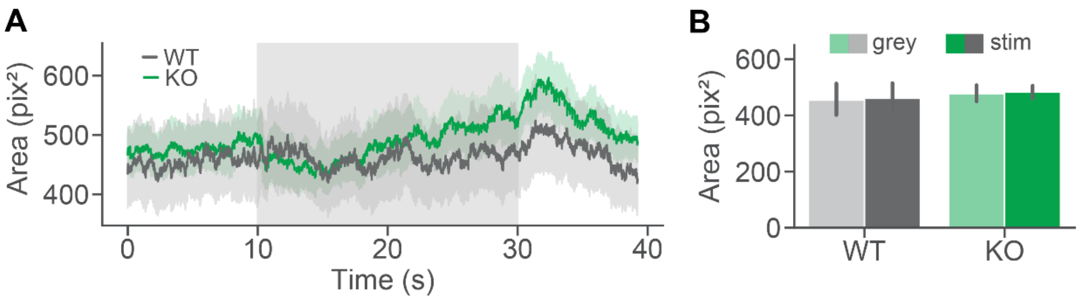

Figure S16. Comparable pupil size across stimulus presentation between WT and Aux KO mice, related to Figure 8.

(A) Pupil area across the entirety of the trial, including visual stimulus presentation (grey box) and intermediate grey screen for WT (grey) and Aux-KO (green) mice.

(B) Quantification of the average pupil area during grey screen and stimulus presentations. Mann–Whitney U test. WT: N=6 mice, Aux-KO: N=5 mice. Data were presented as mean ± SEM.
